# Accumulation of DNA G‐quadruplex in mitochondrial genome hallmarks mesenchymal senescence

**DOI:** 10.1111/acel.14265

**Published:** 2024-07-02

**Authors:** Kangkang Yu, Feifei Li, Ling Ye, Fanyuan Yu

**Affiliations:** ^1^ State Key Laboratory of Oral Diseases & National Clinical Research Center for Oral Diseases, West China Hospital of Stomatology Sichuan University Chengdu China; ^2^ Key Laboratory of Green Chemistry and Technology (Ministry of Education), College of Chemistry Sichuan University Chengdu China; ^3^ Key Laboratory of bio‐Resources and eco‐Environment (Ministry of Education), College of Life Sciences Sichuan University Chengdu China; ^4^ Department of Pediatric Dentistry West China Hospital of Stomatology, Sichuan University Chengdu China; ^5^ Department of Endodontics, West China Hospital of Stomatology Sichuan University Chengdu China

**Keywords:** biomarker, G‐quadruplex, mesenchymal stem cell, mitochondrial genome, senescence

## Abstract

Searching for biomarkers of senescence remains necessary and challenging. Reliable and detectable biomarkers can indicate the senescence condition of individuals, the need for intervention in a population, and the effectiveness of that intervention in controlling or delaying senescence progression and senescence‐associated diseases. Therefore, it is of great importance to fulfill the unmet requisites of senescence biomarkers especially when faced with the growing global senescence nowadays. Here, we established that DNA G‐quadruplex (G4) in mitochondrial genome was a reliable hallmark for mesenchymal senescence. Via developing a versatile and efficient mitochondrial G4 (mtG4) probe we revealed that in multiple types of senescence, including chronologically healthy senescence, progeria, and replicative senescence, mtG4 hallmarked aged mesenchymal stem cells. Furthermore, we revealed the underlying mechanisms by which accumulated mtG4, specifically within respiratory chain complex (RCC) I and IV loci, repressed mitochondrial genome transcription, finally impairing mitochondrial respiration and causing mitochondrial dysfunction. Our findings endowed researchers with the visible senescence biomarker based on mitochondrial genome and furthermore revealed the role of mtG4 in inhibiting RCC genes transcription to induce senescence‐associated mitochondrial dysfunction. These findings depicted the crucial roles of mtG4 in predicting and controlling mesenchymal senescence.

## Graphical abstract

Authors developed an mtG4‐specific fluorescent probe (TPA‐mTO) with near‐infrared emission and excellent photostability that enables real‐time detection and long‐term tracking of mtG4 in both living and fixed cells. Utilizing TPA‐mTO, authors for the first time found the significantly role of mtG4 in MSCs senescence.

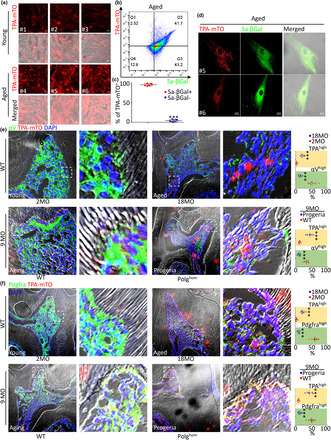

**Abbreviations**
G4: G‐quadruplex
MSC: mesenchymal stem cells
mtDNA: mitochondrial DNA
mtG4: mitochondrial DNAG‐quadruplex
OXPHOS: oxidative phosphorylation
Polg: DNA polymerase g
RCC: respiratory chain complex
Sa‐β‐gal: senescence‐associated β‐galactosidase

## 1 INTRODUCTION

The huge demand for biomarkers of senescence, including the types of healthy chronological senescence, progeria, replicative senescence, and so on, remains largely unmet (Aging Biomarker Consortium et al., 2023; Carlos et al., 2023). Current knowledge of senescence reveal that its hallmarks are mainly consisted of 12 aspects, including mitochondrial dysfunction and genomic instability (Carlos et al., 2023). In addition to the host genomic instability, recent advances also revealed the resurrection of endogenous retroviruses reinforced human senescence (Carlos et al., 2023). These experimental advances have provoked the development of quite a few methods of detecting senescence in vitro and in vivo (Aging Biomarker Consortium et al., 2023; Carlos et al., 2023); however, even with these many advances emerged, evaluating of senescence is still challenging for many limitations of current biomarkers (Aging Biomarker Consortium et al., 2023; Carlos et al., 2023; Fedor et al., 2020; Luise et al., 2023). Therefore, searching for novel biomarkers identifying senescence, especially for multiple types of senescence, is still of great importance (Aging Biomarker Consortium et al., 2023; Carlos et al., 2023; Fedor et al., 2020; Luise et al., 2023).

Genomic instability can be divided into three parts as nuclear DNA, nuclear architecture, and mitochondrial DNA (mtDNA) (Carlos et al., 2023). Compared with the progress of nuclear DNA instability and nuclear architecture, researches on mitochondrial genomic instability and senescence are far lagged (Aging Biomarker Consortium et al., 2023; Carlos et al., 2023; Fedor et al., 2020; Luise et al., 2023). Somatic mutations and deletions of mtDNA often happened due to the high replicative index, the limited efficiency of repair mechanisms, oxidative microenvironment, and the lack of protective histones embracing this small DNA molecule (Carlos et al., 2023; Monica & Scott, 2022). Direct evidences of mtDNA instability being involved in in vivo senescence have been reported by studies which generated the deficient proof reader of mtDNA replication, namely mutant DNA polymerase g (Polg^D257A^) (Aleksandra et al., 2004; Kujoth et al., 2005; Monica & Scott, 2022; Raymond et al., 2011). Polg^D257A^, rendering the expressed mutant protein devoid of polymerase proofreading function in mitochondria, causes the homozygous mutator mice develop progeria‐like premature senescence across organs and a∼50% reduction in lifespan via increasing instability of mitochondrial genome (Aleksandra et al., 2004; Kujoth et al., 2005; Monica & Scott, 2022; Raymond et al., 2011). Specifically, homozygous Polg^D257A^ mutation substantially increased the frequency of multiple types of mtDNA mutagenesis including point mutations, small indels, large deletions, and so on, finally causing mitochondrial dysfunction (Aleksandra et al., 2004; Kujoth et al., 2005; Monica & Scott, 2022; Raymond et al., 2011). The increased mitochondrial genomic instability by homozygous Polg^D257A^ finally caused reduced oxidative phosphorylation (OXPHOS) but aggravated apoptosis in vivo (Aleksandra et al., 2004; Kujoth et al., 2005; Monica & Scott, 2022; Raymond et al., 2011). These results indicate that certain types of somatic mtDNA mutation can lead to mitochondrial dysfunction to speed up senescence progression. However, up to date there is no detectable and feasible senescence‐associated biomarkers discovered from mitochondrial genome. For this question we identified and characterized the DNA G‐quadruplex (G4) accumulating in mitochondrial genome during senescence as a hallmark for mesenchymal senescence. We utilized multiple models of senescence including chronological healthy senescence, progeria, and replicative senescence in this study, which all verified the sensitivity and reliability of mtG4 accumulation as the core hallmark of mesenchymal stem cells (MSC) senescence.

G4s are cation‐stabilized secondary structures that formed by guanine‐rich nucleic acid, which have been proven to be involved in the transcription, recombination and replication of nuclear genome function (Cahoon & Seifert, 2009; Chambers et al., 2015; Rhodes & Lipps, 2015). With respect to the function of G4s on regulating mtDNA, only a very recent study revealed that mtDNA G4 (mtG4) structures influenced both replication and transcription of mitochondrial genes, thus affecting the mitochondrial respiration in mouse embryonic fibroblast cell lines (Falabella et al., 2019). As a semi‐autonomous energy factory in cells, mitochondria only contain a small genome, mtDNA, that encodes the critical genes required for mitochondrial respiration (Giacomello et al., 2020). In this study, we established that accumulation of mtG4 in this small genome was steadily increased within specific mtDNA locations governing respiratory chain complex (RCC) I and IV. Furthermore, we revealed the mtG4 accumulation in MSC senescence contributing to mitochondrial dysfunction via impairing transcription of G4‐enriched RCC I and IV genes.

It remains very difficult and challenging to detect and monitor the extremely tiny special structures of mtDNA, like mtG4 (Chambers et al., 2015; Falabella et al., 2019; Rhodes & Lipps, 2015). Therefore, due to the shortage of live‐cell and intra‐mitochondria imsenescence tools to dynamically exhibit the molecular and structural alterations of mtG4 no previous studies are capable of elucidating mtG4 functions both in vivo and in vitro (Chambers et al., 2015; Falabella et al., 2019; Rhodes & Lipps, 2015). To study the role and regulatory mechanisms of mtG4 in mesenchymal senescence, a brand‐new mtG4 probe (TPA‐mTO) with near‐infrared emission, ideal photostability, and high fluorescence contrast was developed in this study, which is the efficient and reliable tool to visualize mtG4 both in vivo and in vitro, both in living and fixed tissue/cells.

In summary, via developing a versatile and reliable mtG4 probe we revealed that accumulation of DNA G4 in mitochondrial genome hallmarks mesenchymal senescence. mtG4 in aged MSC inhibited RCC I and IV transcription to impair mitochondrial respiration. Our findings demonstrated the detectable and steady senescence‐associated biomarker from mitochondrial genome, and endowed us with brand new insights of mtG4 on regulating mesenchymal senescence.

## 2 RESULTS

### 2.1 Development of feasible and reliable detecting probe for mtG4

**FIGURE 1:**
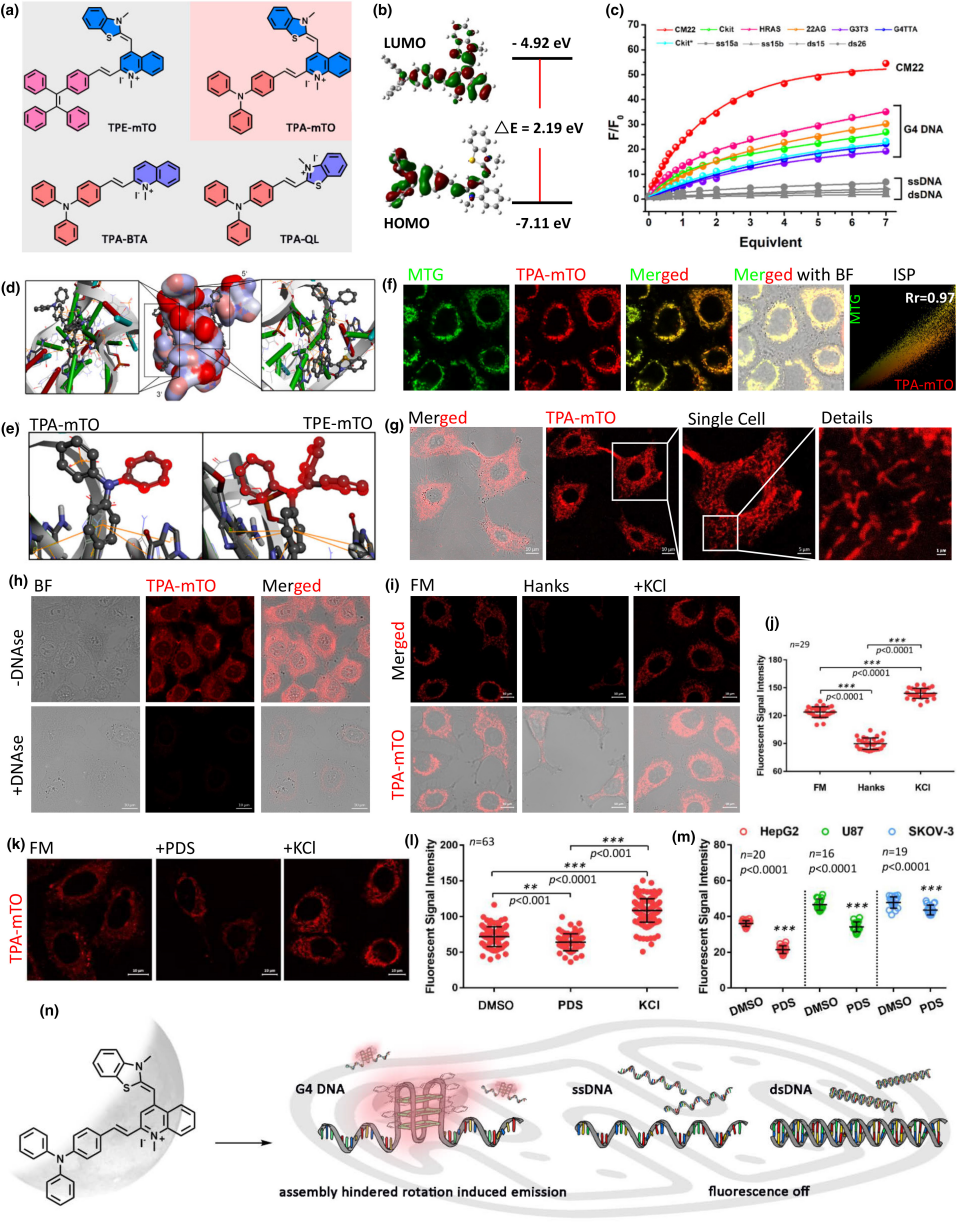
Structures and fluorescent properties of the newly developed probes. (a) The structure of TPE‐mTO, TPA‐mTO, TPA‐BTA and TPA‐QL. (b) Molecular orbital amplitude plots of HOMO and LUMO energy levels of TPA‐mTO calculated through B3LYP/6‐31G (d, p) basis set. (c) The fluorescent intensity of 1 μM TPA‐mTO at 650 nm against the ratio of [nucleic acid sample & probe]/[probe], the testing buffer is 10 mM Tris–HCl (pH 7.4, containing 50 mM KCl), λex = 488 nm. (d) The calculated binding mode of TPA‐mTO with CM22 (G4) via docking study (PDB entry 2l7v). (e) The enlarged view of TPA‐mTO and TPE‐mTO binding to 5′ binding pocket of CM22(G4), respectively. (f) The distributions of TPA‐mTO in A549 cells. The cells were stained with 1 μM TPA‐mTO for 15 min and wish with PBS, then incubated the cells with 1 μM MitoTracker Green (MTG) 20 min. (g) Confocal fluorescent images of A459 cells stained with 1 μM TPA‐mTO for 15 min. (h) Representative confocal fluorescent images of A549 cells stained with 1 μM TPA‐mTO for 15 min without and with DNase treatment. (i) Representative confocal fluorescent images of A549 cells that respectively treated with hanks buffer and KCl and then stained with 1 μM TPA‐mTO for 15 min. (j) Quantification of the fluorescence enhancement caused by the binding between TPA‐mTO and mtG4 in i. (k) Representative confocal fluorescent images of A549 cells that respectively treated with PDS and KCl and then stained with 1 μM TPA‐mTO for 15 min. (l) Quantification of the fluorescence enhancement caused by the binding between TPA‐mTO and mtG4 in k. (m) Quantification of the fluorescence enhancement caused by the binding between TPA‐mTO and mtG4 in different cells with or without PDS. (n) The scheme of selectively detecting mtG4 with TPA‐mTO. Ex@543 nm excitation for the red channel of TPA‐mTO (Em@600–700 nm). Ex@488 nm excitation for the green channel of TPE‐mTO (Em@500–600 nm). Ex@488 nm excitation for the green channel of (Em@500–530 nm). **p < 0.01; ***p < 0.001; no marks indicated no statistical significance.

Inspired by aggregation‐induced emission (AIE) process, we developed a zero‐background mtG4 probe (TPA‐mTO) based on the strategy of fluorescence emission that induced by assembly hindered rotation. (Figure 1a, Extended Data Scheme1, Figure S1) (Wang et al., 2015). TPA‐BTA, TPA‐QL, and previously reported TPE‐mTO were also prepared to compare the differences of D‐π‐A system. As displayed in Figure 1b and Extended Data Figure 1a, the electron distributions of the highest and the lowest occupied molecular orbital (HOMO and LUMO) for TPA‐mTO and TPE‐mTO were similar, which respectively located on the rotatable skeleton and the quinoline moiety, indicating an electron transfer within the two compounds. In addition, the energy gape of TPA‐mTO was larger than that of TPE‐mTO, which means TPA‐mTO will exhibit longer emission wavelength and more obvious fluorescence contrast after biding with G4. As for TPA‐BTA and TPA‐QL, which with simpler shapes, the electrons of both HOMO and LUMO are almost distributed among the whole molecule (Extended Data Figure 1a), suggesting there were no obvious charge transformation within the two molecules, which also means the interaction of DNA bases with TPA‐BTA and TPA‐QL will not result in obvious fluorescence changes.

To confirm the selective fluorescence response of these compounds towards G4, especially TPA‐mTO, the fluorescence spectra of these compounds with various types of nucleic acid were examined (The sequences of nucleic acids were listed in Extended Data Table 1). As shown in Figure 1c, the fluorescence enhancements of TPA‐mTO that induced upon the interaction with G4 (around 20‐fold to 50‐fold) were much more significant compare to the enhancements caused by the addition of ssDNA and dsDNA (less than five‐fold), indicating TPA‐mTO did exhibit G4 selective affinity and showed no preference for any particular G4 conformation (Figure S2). The quantum yield of TPA‐mTO was close to 0, while the value increased to 7% when the molecule was bound to prefolded G4 (CM22). For TPA‐BTA and TPA‐QL, although the gradual addition of G4 induced an increase of their fluorescence, it was hard to clearly distinguish G4 from ssDNA and dsDNA based on the fluorescence enhancement, since the difference of fluorescence enhancements caused by G4 and ssDNA/dsDNA was not obvious (Extended Data Figure 1b,c; Figure S3,S4). We also verified that TPA‐mTO would not affect the topology of G4 structure by testing the circular dichroism (CD) spectra of each oligonucleotide sequence in the presence of TPA‐mTO (Figure S5). The binding stoichiometry, apparent binding constants (Ka), detection limits (LOD), and linear detection range of TPA‐mTO towards different G4 were also investigated and the results demonstrated that TPA‐mTO is a feasible and reliable tool for detecting G4 (Table 1; Figure S6‐S8), which was consistent with the frontier molecular orbital analysis.

**TABLE 1:** The binding stoichiometry, apparent binding constants (K_a_), detection limits (LOD), and linear detection range of TPA‐mTO towards different G‐quadruplexes. The concentration of TPA‐mTO is 1 μM and the testing buffer is 10 mM Tris–HCl (pH 7.4, containing 50 mM KCl).

Name of G4 DNA	Stoichiometry (TPA‐mTO:DNA)	K_a_[10^6^ M^−1^]	LOD (μM)	LDR (μM)
CM22	2:1	4.83	0.02	0–0.6
Ckit	2:1	2.18	0.04	0–0.6
HRAS	1:1	0.53	0.04	0–1.0
22AG	1:1	0.27	0.06	0–1.6
G3T3	2:1	0.41	0.12	0–2.0
G4TTA	1:1	0.14	0.09	0–2.0
Ckit*	1:1	0.19	0.14	0–2.0

To explore the interaction between DNA bases and TPA‐mTO, the binding mode of TPA‐mTO in CM22 was investigated. As depicted in Figure 1d, bimolecular TPA‐mTO were respectively docked into the probe‐induced binding pockets at both 3′ and 5′ flanking sequences of CM22 via π‐π interaction, π‐cation interaction, and hydrogen bonds. Compared with our previously reported TPE‐mTO, although the number of interaction bonds did not change much, the range of probe‐G4 interaction sites altered greatly (Figure 1e), which explained the reason why the fluorescence enhancement of TPA‐mTO (54‐fold) caused by G4 was much more obvious than that of TPE‐mTO (23‐fold).

On the basis of above reported capabilities of TPA‐mTO to recognize G4 structures in vitro, we furthermore verified its efficacy and safety of mtG4 imsenescence in living cells. The probe labelling data showed no obvious side effect on cell viability (Extended Data Figure 1d). Then, the co‐locations results showed that the red channel of TPA‐mTO overlapped pretty well with the MTG channel but matched improperly with the channel of LTG (Rr were respectively calculated as 0.97 and 0.46, Fig. f, Extended Data Figure 1f), implying TPA‐mTO showed selective binding affinity to G4 structures in mitochondria. Meanwhile, the experiments of TPA‐BTA and TPA‐QL in A549 cells showed pretty low Pearson's correlations with MTG channel (Rr not exceed 0.85), suggesting the two compounds with simpler molecular shapes could not effectively image mtG4. The confocal fluorescent images of TPA‐mTO incubated cells all displayed strong fluorescence signal in certain regions of cytoplasm and the signal displayed solid short rod‐like shapes with different intensities (Figure 1g, Extended Data Figure 2a), suggesting TPA‐mTO selectively bound with G4 structures in mitochondrial matrix of live cells. The deoxyribouclease (DNase) digestion, Hanks treatment, and KCl treatment experiments were successively conducted and proved that the DNA structures, selectively bound by TPA‐mTO and stabilized by potassium ion in mitochondrial matrix of live cells, were mtG4 (Figure 1h–j). Next, we assessed that TPA‐mTO, unlike previously reported TPE‐mTO, still exhibited excellent mtG4 binding affinity in fixed cells with excellent photostability (Extended Data Figure 2b,c). We also found that TPA‐mTO stained live cells still showed good imsenescence results after 48 hours, indicating our probe also possessed long term imsenescence capability (Extended Data Figure 2d,e). Taken together, these data demonstrated that TPA‐mTO was a promising tool for dynamic mtG4 imsenescence with excellent photostability and long‐term tracking capability. Moreover, it was also proved via TPA‐mTO that KCl instead of PDS (a heavily‐utilized G4 stabilizing agent (Biffi et al., 2013)) was an effective mtG4 agonist in various cell types (Figure 1k–m and Extended Data Figure 2f), therefore KCl was used to modulate mtG4 contents in following procedures.

**FIGURE 2:**
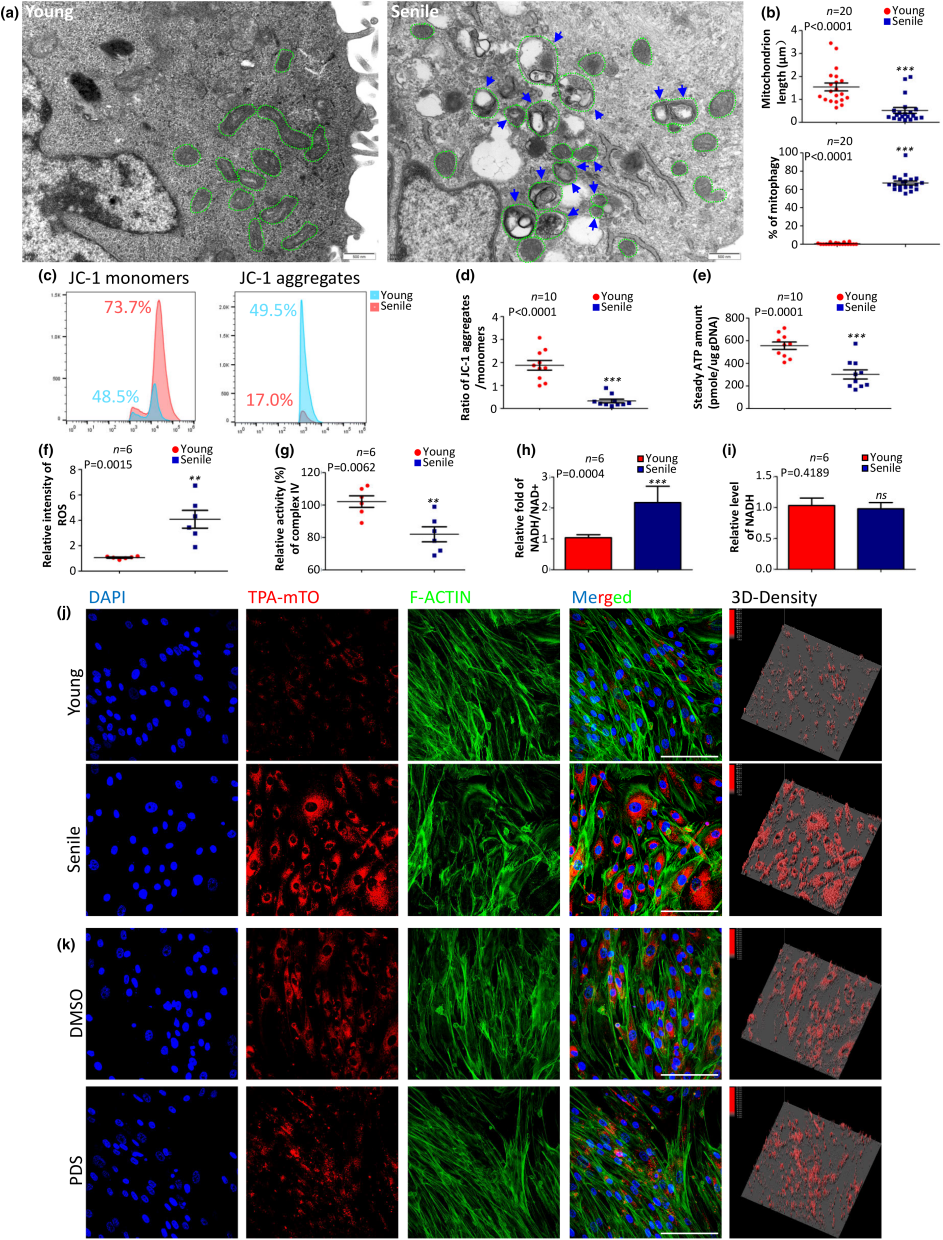
Evidences of MSC senescence and mitochondrial dysfunction in Senile MSC and TPA‐mTO‐based mtG4 imsenescence of MSC. (a) TEM images of young and senile MSC. The green dotted circles showed the contours of mitochondria, and the blue arrows indicated the occurrence of mitophagy. Scale bars, 500 nm.(b) Statistic data of the mitochondria length (top) and the percentages of mitophagy in total amounts of mitophagy per cell (bottom). (c) Representative images of fluorescence‐activated flowcytometry analysis (FACs) using JC‐1 probes. (d) Statistic data of the ratio of JC‐1 aggregates versus monomers between young and senile MSC. (e) The steady ATP amount between young and senile MSC. (f) Statistic data of ROS level between young and senile MSC. Data were obtained via FACs using ROS probes. (g) Statistic data of the relative activity of respiratory chain complex IV using the previously reported detection methods ^38^. (h) Statistic data showing the ratio of NADH versus NAD^+^ concentrations between young and senile MSC. (i) Statistic data of the relative level of NADH between young and senile MSC. (j) Representative confocal fluorescent images of TPA‐mTO‐labeled young and senile MSC. Cells were counterstained using DAPI and phalloidin. The 3‐dimension fluorescence density (3D‐density) was calculated via image pro plus (IPP). Scale bars, 15 μm. (k) Representative confocal fluorescent images of TPA‐mTO‐labeled young MSC and PDS‐pretreated young MSC. Cells were counterstained using DAPI and phalloidin. The 3D‐density was calculated via image pro plus (IPP). Scale bars, 15 μm. **p < 0.01; ***p < 0.001; no marks indicated no statistical significance.

From the characterization of G4 probes, we could confirm that (1) obvious and effective D‐π‐A system was critical to construct G4 probes with significant fluorescence contrast; (2) the molecules with a certain angle would have better G4 affinity (Xu, 2013); (3) the appropriate amount of charge and size of molecules were essential factors that affected the selective binding of mtG4. These results proved TPA‐mTO as a promising candidate for selective recognition of mtG4, which exhibited low background, obvious fluorescence contrast, and near‐infrared emission during the process of mtG4 recognition (Figure 1n).

### 2.2 mtG4 hallmarked replicative senescence of MSC

Our recent findings showed that replicative senescence significantly impaired the stem/precursor characteristics and regenerative capabilities of human dental pulp derived MSC (Lin et al., 2023). Therefore, in this study we first used TPA‐mTO to investigate if mtG4 was involved in replicative senescence of MSC. The MSC mentioned in this study referred to dental pulp‐derived MSC, otherwise specifically indicated.

We established the replicative senescence model of human MSC (Extended Data Figure 3a,b) following our previous publication (Lin et al., 2023). Transmission electron microscopic (TEM) data showed that replicative senescence severely broken the morphological normality of mitochondria (Figure 2a). Almost all mitochondria in replicative senescence group became shortened and round, showing standard alterations of mitophagy (Figure 2a). Statistical results quantitatively demonstrated the significantly increased incidence of mitophagy and reduced mitochondria length in replicative senescence group (Figure 2b). Next, flow cytometry (FC) data of JC‐1 showed the mitochondrial membrane potential substantially descended in replicatively aged MSC (Figure 2c,d). Following the decreased membrane potential, we next discovered that replicatively aged MSC showed decreased steady ATP (Figure 2e) but increased reactive oxygen species (ROS) (Figure 2f). These data together indicated severe mitochondrial dysfunction happened in replicative senescence of MSC. We found out that activity of RCC I and IV were remarkably impaired in replicative senescence group (Figure 2g–i). Using this senescence model and TPA‐mTO, we next detected the alterations of mtG4 during replicative senescence of MSC. Results showed that in replicatively senescent MSC, the contents of mtG4 were substantially increased (Figure 2j). Similar to our findings observed in other types of cells (Extended Data Figure 2f), PDS did not change mtG4 contents in MSC (Figure 2k), furthermore supporting the infeasibility of utilizing PDS to regulate mtG4 formation and proving the accuracy of mtG4 accumulation hallmarking replicative senescence of MSC.

**FIGURE 3:**
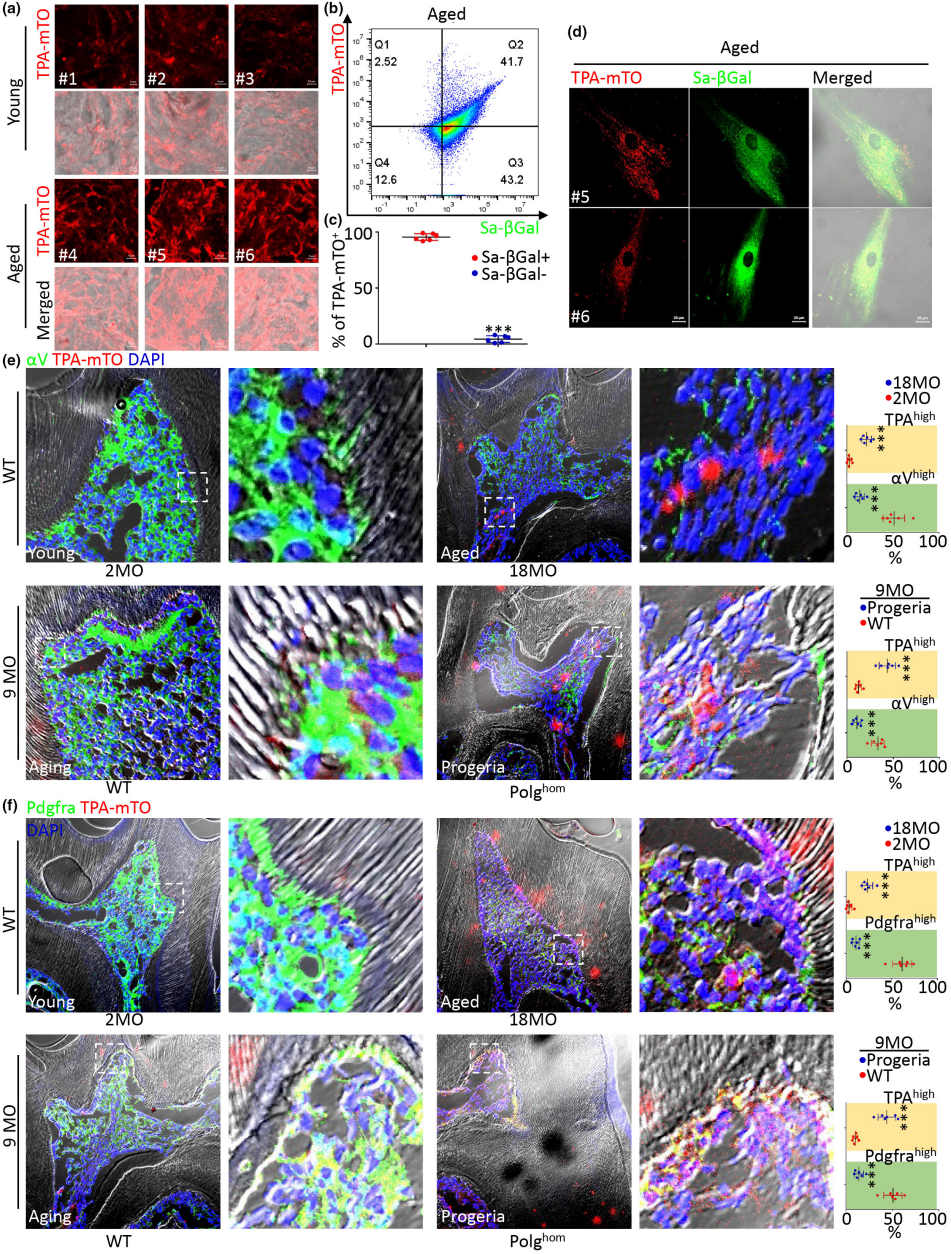
mtG4 Hallmarked Chronological and Progeria‐associated MSC Senescence. (a) Representative TPA‐mTO imsenescence data of human dental pulp of healthy third molar mesenchyme, merged in bright field. Young group: #1‐#3, three healthy individuals aged as 18yo, 22yo, and 21yo; Aged group: #4‐#6, three healthy individuals aged as 78yo, 72yo, and 81yo. Scale bars, 5 μm. (b, c) Representative FC results of aged MSC (b) and its statistic data (c). *n* = 6 individuals. (d) Representative fluorescence images of aged MSC from aged donor #5 and #6. Scale bars, 20 μm. (e, f) Representative fluorescence images of MSC (left) and its statistical data (right). MO, moths‐old; WT, wild type; Polg^hom^, homozygotes of Polg^D257A^ mutators. For statistical analysis, *n* = 6 individuals. Scale bars, 15 μm. ***p < 0.001.

### 2.3 mtG4 hallmarked chronological and progeria‐associated MSC senescence

In addition to replicative senescence, we next investigated the feasibility of using mtG4 as the senescence‐associated biomarker in other types of MSC senescence. Data of human dental pulp tissues showed the contents of mtG4 being notably increased in healthy chronological aged individuals, causing most MSC in aged individuals were mtG4 highly‐accumulated (Figure 3a). Furthermore, CF results quantitively showed that near 100% mtG4^+^ MSC were senescence‐associated β‐galactosidase (Sa‐β‐gal)^+^ in chronologically aged humans (Figure 3b,c). Confocal imsenescence in details showed the high correlation between mtG4 accumulation and chronological senescence of MSC (Figure 3d). Finally, using transgenic mouse models we verified the reliability of mtG4 hallmarking chronological and progeria‐associated MSC senescence (Figure 3e,f). Data showed that both chronological senescence and progeria demonstrated reduced Pdgfra+ and Cd51 expressions in MSC, showing the senescence of mesenchymal tissues, but mtG4 contents were substantially increased (Figure 3e,f). Together, our data revealed that mtG4 hallmarked chronological and progeria‐associated MSC senescence.

### 2.4 Potassium ions controlled mtG4 formation in MSC

**FIGURE 4:**
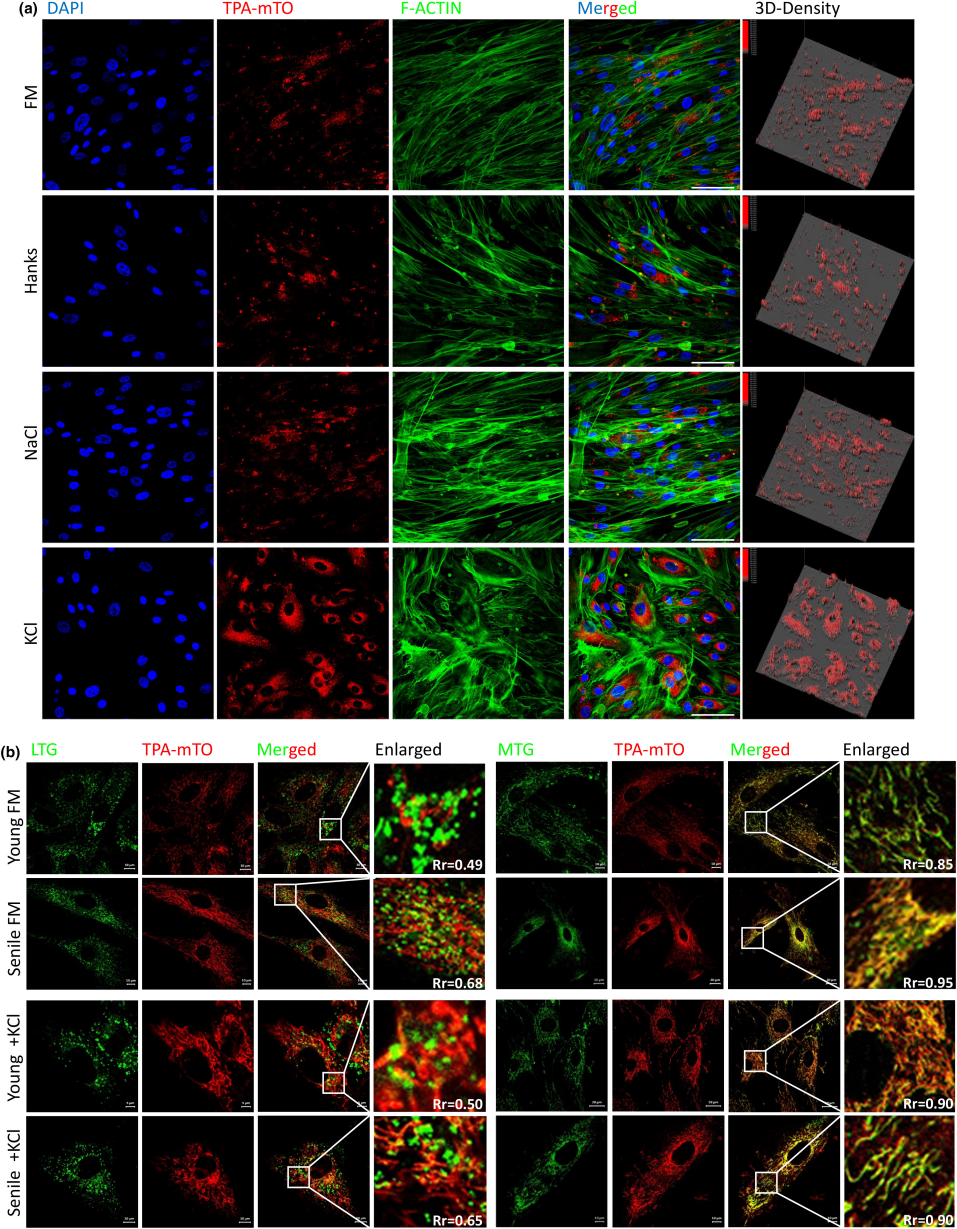
TPA‐mTO‐based imsenescence of K^+^ controlled mtG4 formation in MSC. (a) Representative confocal fluorescent images of TPA‐mTO‐labeled young and senile MSC, which were respectively treated with full medium (FM), K^+^ and Mg^2+^‐free hanks buffer, 200 mM NaCl and 200 Mm KCl. Cells were counterstained using DAPI and phalloidin. The 3D‐density was calculated via IPP. Scale bars, 15 μm. (b) Representative confocal fluorescent images of young and senile MSC that respectively treated with full medium (FM) and KCl, then co‐stained with Lyso Tracker Green (LTG)/Mito Tracker Green (MTG) and TPA‐mTO. The Pearson's coefficients were all listed in the images. Ex@543 nm excitation for the red channel of TPA‐mTO (Em@600–700 nm). Ex@488 nm excitation for the green channel of LTG and MTG (Em@500–530 nm). Scale bars, 10 μm.

After acknowledging the feasibility and reliability of mtG4 as the senescence marker of MSC, we next sought to investigate the function and mechanisms of mtG4 underlying MSC senescence. For this objective we identified a regulatory tool of mtG4 in MSC which was potassium ions (K^+^) (Figure 4). As PDS cannot regulate mtG4 in MSC (Figure 2k), we detected another known regulator of G4, namely K^+^. Results showed that in MSC the addition of K^+^ notably increased mtG4 contents, and this effect was not affected by osmotic stress (Figure 4a). In combination of using standard mitochondria probe, mitotracker (MTG) we again proved the accuracy of TPA‐mTO on imsenescence mitochondrial sub‐organelle structure (Figure 4b). Besides, K^+^ increased mtG4 contents in young MSC, but due to the baseline was already very high in aged MSC K^+^ did not additively cause notably more mtG4 (Figure 4b). In young MSC, very low correlation between mtG4 and lysosome activity was observed (Figure 4b), showing limited mitophagy in this condition. But for aged MSC those mtG4‐accumulated mitochondria demonstrated up‐regulated mitophagy (Figure 4b). With adding of K^+^ young MSC also demonstrated increased mitophagy in those mtG4‐accumulated mitochondria (Figure 4b). These data together revealed that K^+^ was capable of controlling mtG4 formation in MSC, thus in the following experiments we used K^+^ to analyze the function and underlying mechanisms of mtG4 in MSC senescence.

### 2.5 Sub‐organelle characterization of mtG4 in MSC senescence

**FIGURE 5:**
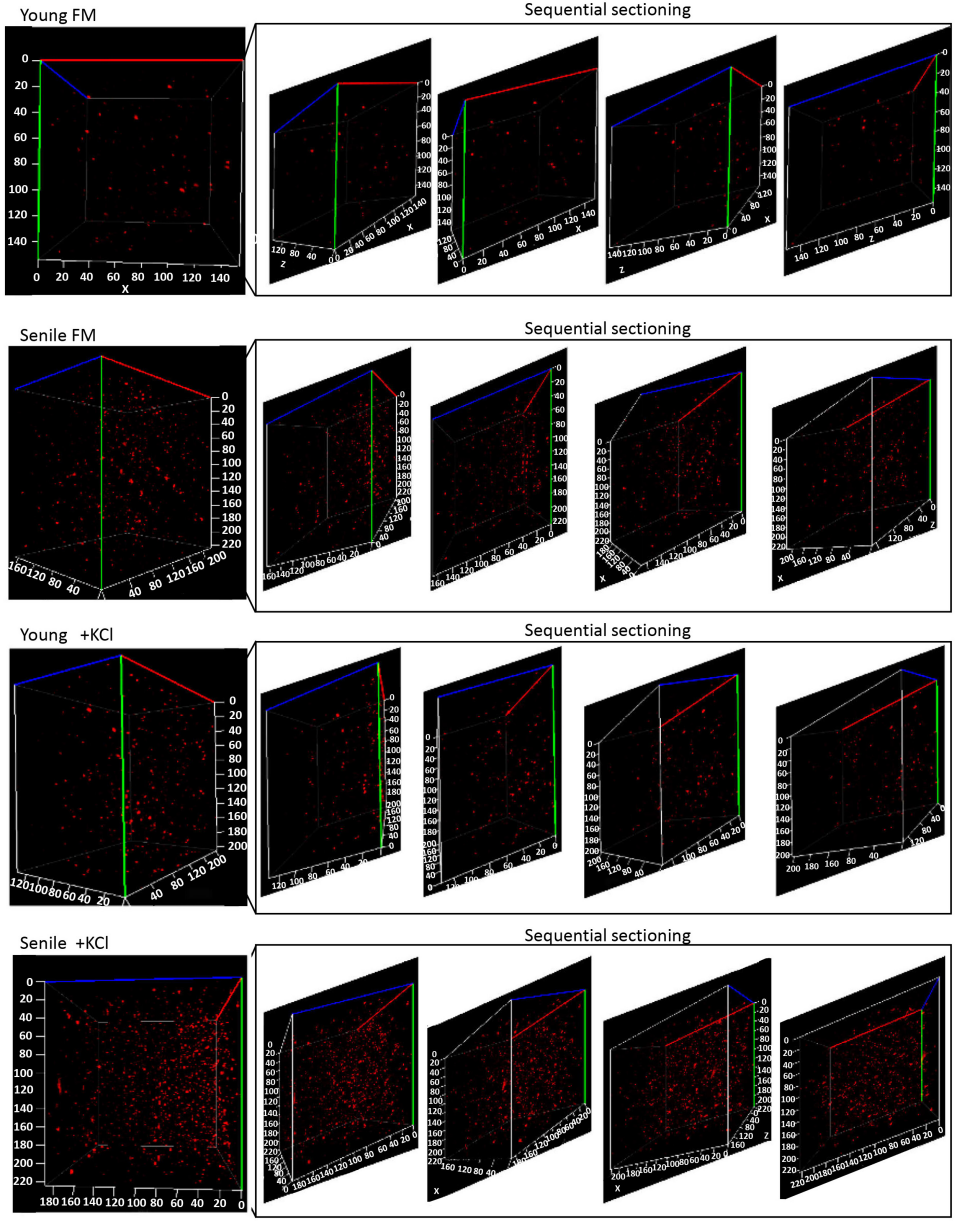
Representative confocal fluorescent images of TPA‐mTO‐labeled freshly isolated mitochondria from young and senile MSC, which were respectively treated with FM or 120 mM KCl before detection. 3D confocal images of the same volume of active mitochondria stained with TPA‐mTO. Each group showed the same numbers of cells‐derived mitochondria.

To visualize mtG4 contents in sub‐organelle level, we isolated mitochondria from both young and aged MSC with the same cell numbers (Figure 5). These isolated fresh mitochondria were probed using TPA‐mTO. Data showed that in sub‐organelle layer, aged MSC‐derived mitochondria owned much more mtG4 (Figure 5), which was consistent with the results observed in cellular layer. Via treating mitochondria isolated from young MSC with K^+^, we discovered remarkably increased mtG4 numbers and intensity to the extent equaling with that of aged MSC‐derived mitochondria (Figure 5). This result provided direct evidence that K^+^ efficiently up‐regulated mtG4 formation in MSC. Treating mitochondria isolated from aged MSC with K^+^ we furthermore observed more mtG4 contents either than aged MSC‐derived mitochondria without K^+^ treatment or than young MSC‐derived mitochondria with K^+^ treatment (Figure 5). It demonstrated that in sub‐organelle level K^+^ treatment additively increased mtG4 contents even in aged MSC, which recompensed the limitation of information acquisition in cellular layer (Figure 4b).

### 2.6 Accumulated mtG4 impaired RCC I and IV transcription in MSC senescence

**FIGURE 6:**
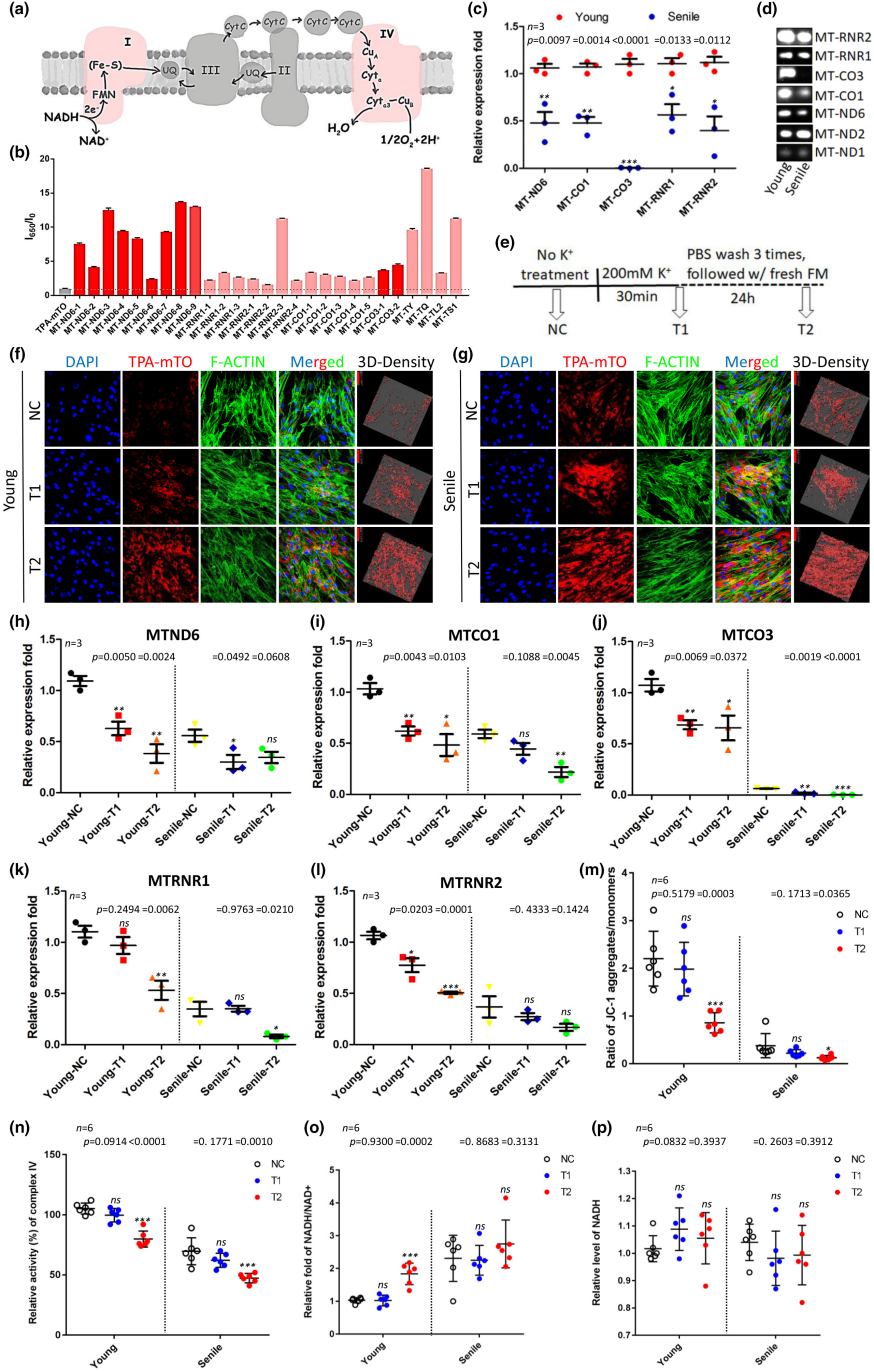
mtG4 represses mtDNA transcription in senile MSC. (a) The schematic illustration of mitochondria respiratory chain. Complex I and IV were highlighted in red. (b) The intensity changes of TPA‐mTO before and after addition of various selected oligonucleotides of mtDNA in 10 mM Tris–HCl buffer containing 50 mM KCl. (c) RT‐qPCR data of mRNAs from isolated mitochondria. The expression level of each gene was normalized to MT‐ND2. (d) The representative images of agarose gel electrophoresis showing the gene expressions of isolated mitochondria. (e) The schematic illustration of experimental procedures that were followed from Figure 5f–p. (f) TPA‐mTO detection showed increased mtG4 level in young MSC after 200 mM KCl treatment. (g) TPA‐mTO detection showed increased mtG4 level in senile MSC after 200 mM KCl treatment. (h)‐(l) RT‐qPCR data of mRNAs from isolated mitochondria following the experimental procedure in Figure 5e. The expression level of each gene was normalized to MT‐ND2. (m) Statistic data of the ratio of JC‐1 aggregates versus monomers between young and senile MSC after 200 mM KCl treatment. (n) Statistic data of the relative activity of respiratory chain complex IV using the previously reported detection methods^38^. (o) Statistic data showing the ratio of NADH versus NAD^+^ concentrations between young and senile MSC after 200 mM KCl treatment. (p) Statistic data of the relative level of NADH between young and senile MSC after 200 mM KCl treatment.

As we proved the reliability of mtG4 as the hallmark of MSC senescence, we next investigated the cellular and molecular mechanisms underlying mtG4‐associated regulations in MSC senescence. Using in‐silicon screening and scoring we found the most putative mtDNA genes which may form mtG4 structures. These mtDNA sequences were furthermore analyzed via QGRS Mapper, thus finally we got the sequences that might form G4 structures among these genes (listed in Extended Data Table 1). Next, in vitro we used TPA‐mTO to verify the possibilities of G4 structure formation in these QGRS Mapper‐selected mtDNA sequences, and their CD spectra were tested in 10 mM Ttris‐HCl buffer containing 50 mM KCl (Figure S9). The CD spectra of the selected mtDNA sequences in buffer containing KCl depicted a negative peak around 240 nm and a positive peak around 270 nm, while no peaks showed up in water, implying the selected mtDNA fragments could form G4 structures in the presence of K^+^. mtDNA genes, MT‐CO1, MT‐CO3, and MT‐ND6, were proved to be theoretically capable of forming multiple G4 structures (Figure S9). As shown in our schematic illustration, these three genes were crucial for respiratory chain functions in mitochondria (Figure 6a). We next confirmed the fluorescence response of TPA‐mTO towards the selected mtDNA fragments. Data showed that the fragments of mtDNA caused notable fluorescence enhancement of TPA‐mTO, indicating mtG4 was easily formed mainly within these specific mtDNA locations governing RCC I and IV genes (Figure 6b). After in vitro screening of candidate sites for mtG4 accumulation, we investigated the roles of mtG4 in transcription of these mtG4‐enriched genes and their functions on regulating MSC senescence. RT‐qPCR results of isolated fresh mitochondria showed the expressions of certain high‐mtG4 enriched genes identified in vitro were significantly decreased in senile MSC, including MT‐CO1, MT‐CO3, MT‐ND6, MT‐RNR1, and MT‐RNR2 (Figure 6c,d). In accordance to both in‐silicon scoring and preliminary assessment data (Figure 6c,d, Extended Data Table 1; Figure S9), we did not find the high possibility of forming mtG4 structures within MT‐CO2. RT‐qPCR results furthermore confirmed that no transcriptional alterations of MT‐CO2 happened in senile MSC‐derived mitochondria (Figure 6d). Therefore, MT‐CO2 was used as the reference gene in our following RT‐qPCR experiments to normalized mtDNA expression in this study. To functionally verify whether the increased mtG4 contents in senile MSC directly inhibit mtDNA transcription, we used K^+^ to manipulate mtG4 levels. To avoid the probably rapid impact of K^+^ on changing mitochondrial membrane potential, we set two timing points of detection (Figure 6e). TPA‐mTO imsenescence data showed that at the timing point 1 (T1) after 200 mM KCl treatment, both young and senile MSC exhibited much higher contents of mtG4 (Figure 6f,g). At the timing point 2 (T2), the upregulation of mtG4 was furthermore enhanced in both young and senile MSC (Figure 6f,g). RT‐qPCR data of isolated fresh mitochondria next showed that the five mtG4‐enriched genes, MT‐CO1, MT‐CO3, MT‐ND6, MT‐RNR1, and MT‐RNR2, were significantly transcriptionally repressed after KCl treatment both in young and senile MSC (Figure 6h–l). Especially, for MT‐CO3 its transcription descended a lot in senile MSC’ mitochondria, and such kind of transcriptional repression was further aggregated even in senile MSC after increasing mtG4 contents via K^+^ treatment (Figure 6j). The same phenomenon was also observed in MT‐ND6 (Figure 6h), MT‐CO1 (Figure 6i), MT‐RNR1 (Figure 6k), and MT‐RNR2 (Figure 6l). Finally, we detected whether or not by inducing mtG4 we could partially phenocopy senescence‐associated mitochondrial dysfunction and impaired respiratory chain. At the T2 point, K^+^‐induced mtG4 was found to decrease the membrane potential in both young and senile MSC (Figure 6m). The main mtG4‐repressed genes found by us, namely MT‐CO1, MT‐CO3, and MT‐ND6, were respectively vital for RCC I and IV (Figure 6a), and accordingly we observed the weakened activities of RCC I and IV in senile MSC (Figure 2f–i). Following these evidences, we next measured the possible functions of mtG4 on impairing RCC I and IV activities. Data consequently proved that the activity of complex IV was significantly damaged at the T2 point after KCl treatment, especially in senile MSC (Figure 6n). As for complex IV function, we found that after we induced mtG4 using KCl, the NADH/NAD^+^ redox decreased both in young and senile MSC at T2, but there lacked statistical significance in senile MSC (Figure 6o,p). Taken together, these data supported the potential roles of mtG4‐orchestrated gene repression in contributing to respiratory chain damage and mitochondrial dysfunction in MSC senescence (Figure 5).

## 3 DISCUSSION

Searching for more reliable and detectable biomarkers of senescence remains very urgent (Aging Biomarker Consortium et al., 2023; Carlos et al., 2023; Fedor et al., 2020; Luise et al., 2023; Monica & Scott, 2022). Although certain biomarkers of senescence have been discovered, the detection of senescence remain challenging (Aging Biomarker Consortium et al., 2023; Carlos et al., 2023; Fedor et al., 2020; Luise et al., 2023; Monica & Scott, 2022). For instance, gene marker p21 and p16 have been reported unreliable for hallmarking senescence in some conditions (Aging Biomarker Consortium et al., 2023; Carlos et al., 2023; Fedor et al., 2020; Luise et al., 2023; Monica & Scott, 2022), canonical Sa‐β‐gal detection can't be dynamically and non‐destructively applied and so on (Aging Biomarker Consortium et al., 2023; Carlos et al., 2023; Fedor et al., 2020; Luise et al., 2023; Monica & Scott, 2022). In this study we presented the first visible senescence biomarker of mesenchyme based on mitochondrial genome instability, which was versatile for multiple types of senescence and can be applied for live, fixed, even formalin‐fixed paraffin‐embedded (FFPE) cells and tissues.

In detail, we revealed that mtG4 hallmarking the genome instability of mitochondria during mesenchymal senescence. Accumulated mtG4 within mtDNA loci of RCC I and IV repressed the transcriptional yield of respiratory mtDNA genes, which directly caused senescence‐associated mitochondrial dysfunction. These findings endow us with new insights on mtG4 functions and underlying mechanisms by which mitochondrial genome instability regulates MSC senescence. Via being measured in multiple models of senescence, including replicative senescence, healthy chronological senescence, and premature progeria, we proved that mtG4 was an accurate hallmark for mesenchymal senescence among various types of senescence.

Up to date no studies report the role of G4 in senescence. In addition to the hallmark function of mtG4 identified in mesenchymal senescence, our data furthermore indicated a possible upstream regulatory mechanism underlying DNA G4 formation in mitochondrial genome. Specifically, it has been discovered that homozygous mutation of Polg^D257A^, rendering impaired mtDNA replication proofreading, caused multiple types of mutagenesis (Aleksandra et al., 2004; Kujoth et al., 2005; Monica & Scott, 2022; Raymond et al., 2011). In this type of progeria‐like premature senescence, we uncovered the accumulated mtG4 in MSC, which showed that replication errors of mitochondrial genome increased mtG4 formation in specific loci such as RCC I and IV. Therefore, the mitochondrial dysfunctions of RCC I and IV in aged MSC theoretically be contributed by mtG4 accumulation. Our data preliminarily proved this point via using K^+^ treatment to upregulate mtG4, revealing the repressive effects of mtG4 on RCC I and IV gene transcription.

We developed TPA‐mTO to be a live‐cell safe and highly mtG4‐specific fluorescence probe, which is the first efficient and reliable tool to analyze the dynamic changes of mtG4 in both live‐ and fixed‐cells and tissues until now. G4‐specific antibodies, namely 1H6 and BG4, are the most utilized agents to detect intra‐cellular G4 structures, but it can only be stained in fixed cells and barely exhibit mitochondrial G4 (Antonio et al., 2020; Liu et al., 2020). In the past few years, the rapid development of fluorescent probes for the specific recognition of G4 has made undisputed progress (Ma et al., 2015; Pandith et al., 2019). However, most of the reported G4 probes are only capable of measuring nuclear G4, it remains challenging to develop more precise and multitasking fluorescent probes that combine mtG4 selectivity/affinity and suitable optical properties (emission wavelengths >600 nm, excellent photostability, low background, and obvious fluorescence contrast). We previously developed a series of efficient fluorescent probes (He et al., 2020; Li et al., 2020; Shi et al., 2020; Yu et al., 2020), among which one probe (TPE‐mTO) selectively bound mtG4 structure within several minutes in live cell (Yu et al., 2020). Unfortunately, the poor photostability and the relatively low fluorescence contrast of TPE‐mTO makes it unsuitable to be used for long‐term tracking and detailed observation with higher resolution. To overcome these limitations, TPA‐mTO, created in this study, provides a general method for analyzing the dynamic changes of mtG4s, enabling the identification of mtG4 contents between young and senile individuals, which can be applied in both alive and dead tissue/cells, even in FFPE tissue specimens. A latest study reported a nucleus‐G4‐specific fluorescent probe, which enabled researchers to uncover that in live cells the amounts of nuclear G4 was cell‐cycle‐dependant (Antonio et al., 2020). In detail, nuclear G4 were mainly formed in G1/S and S phase, thus indicating that the formation of nuclear G4 was possibly associated with nuclear DNA replication (Antonio et al., 2020). Taken our findings of Polg^D257A^ homozygotes together, it implied that both for nuclear genome and mitochondrial genome DNA replication upstream influenced/controlled DNA G4 formation; however, underlying upstream mechanisms of this event remains unknown.

We proved the efficient role of K^+^ but not PDS in triggering mtG4 formation in MSC, with the help of TPA‐mTO we shall in future analysis to screening out more mtG4‐specific inducers and repressors. K^+^ induced the formation of general types of G4, including nuclear and mitochondrial ones for both RNA G4 and DNA G4 (Chambers et al., 2015; Falabella et al., 2019; Rhodes & Lipps, 2015). Therefore, K+ is not the specific regulator for mtG4. The development and identification of mtG4‐specific regulators will help uncover the underlying roles of mtG4. For this purpose, TPA‐mTO is the ideal tool to identify and verify candidate specific regulators for mtG4.

In summary, via developing an efficient and reliable mtG4‐specific fluorescent probe, we identified that accumulation of DNA G4 in mitochondrial genome hallmarked mesenchymal senescence. These findings endowed us with the visible senescence biomarker, mtG4, based on mitochondrial genome instability and furthermore revealed the role of mtG4 in inhibiting RCC genes transcription to induce senescence‐associated mitochondrial dysfunction.

## 4 MATERIALS AND METHODS

Detailed materials and methods were listed in supplementary information.

### 4.1 Experimental animals

All animal studies conformed to ARRIVE (Animal Research: Reporting of In Vivo Experiments) guidelines. C57/B6J mice were purchased from Dossy (Chengdu Dossy Experimental Animals Co., Chengdu, China). Polg^D257A^ were purchased from JAX lab (stock # 017341). With respect to the maintaining of experimental mice, they were housed in specific‐pathogen free Experimental Animal Core of West China Hospital, Sichuan University (China), which was a temperature controlled (25°C) environment under a 12‐hour light/12‐hour dark cycle with cotton batting. To get the homozygous mutant of Polg^D257A^, we followed the guidelines from JAX lab. In brief, the female and male heterozygous Polg^D257A^ mice (that originated from a C57BL/6J female x heterozygous male cross) were crossed to get the homogenous strain.

### 4.2 Calculations of apparent binding constants

The apparent binding constants (K_a_) was calculated based on the following equation^41^.

$$\frac{F}{F_{0}}=1+\frac{Q-1}{2}\left[A+1+x-\sqrt{\left(A+1+x\right)-4x}\right]$$

$$x=n\frac{C_{G-\text{quadruplexe}\;\mathrm{DNA}}}{C_{\mathrm{TPA}-\mathrm{mTO}}}$$

$$A=\frac{1}{K_{a}C_{\mathrm{TPA}-\mathrm{mTO}}}$$

*F*_0_ means fluorescence intensity of TPA‐mTO only at 650 nm, *F* stands for fluorescence intensity at 650 nm of TPA‐mTO with different G4, *n* is the putative number of TPA‐mTO binding to a certain G4. To establish the fitting curve, *Q* and *A* were set up as parameters using the fitting routine in OriginPro 8.5.1 software, whereas *n* was changed to acquire a better fit.

### 4.3 Calculations of detection limit

The detection limit (LOD) was calculated based on the following equation^42^.

$$\mathrm{LOD}=\frac{3\sigma }{k}$$

*σ* means the standard deviation of multiple blank measurements, *k* represents the slope of the calibration curve. (The fluorescence emission of TPA‐mTO was detected 10 times to obtain the standard deviation of blank measurements.)

### 4.4 Molecular docking study

The molecular docking studies were performed using GOLD (Genetic Optimization of Ligand Docking) 5.0. The X‐ray crystal structure (PDB entry 2J6M) of the CM22 G4 bound to compound quindoline was used as a reference in the docking studies.^44^ The Discovery Studio 3.1 (Accelrys, Inc. USA) software package was used to prepare the structure including adding hydrogen atoms, removing water molecules, and assigning force field (here the CHARMm force field was adopted).

### 4.5 Human dental pulp MSC

Primary human dental pulp MSC were harvested and cultured following our previous study (Lin et al., 2023). In brief, all human pulp tissue procedures were reviewed and approved by the Ethical Committees of the West China School of Stomatology, Sichuan University (WCHSIRB‐D‐2020‐393). The primary MSC were passaged upon reaching 80% confluence. To induce senescence of MSC, we used passage 14–16 as senile MSC in this study, and meanwhile cells at passage 2/3 were set as young MSC groups. All senile cells were confirmed via both Sa‐β‐gal staining and EdU incorporation diction before further analysis. For Sa‐β‐gal staining, cells were treated using the Senescence β‐Galactosidase Staining Kit (Beyotime, #C0602) in accordance to manufacturer's protocols. For EdU staining, we used EdU imsenescence kit (RiboBio, China) according to manufacturer's protocol. TEM samples were conducted following standard procedures, and finally detected via JEOL TEM microscope (#JEM‐1400PLUS, Japan). The statistical analysis of TEM was conducted via Image Pro Plus 6.0 (Media Cybernetics, Rockville, MD, USA). Intracellular steady ATP amount measurements were carried out using CellTiter‐Glo kit (Promega, #G7571, USA) according to the manufacturer's instruction, and luminescence was detected via the luminometer of Varioskan Flash (Thermo Scientific, USA). ATP amounts were normalized to total DNA amount in each well. For NADH and NAD^+^ assessments, we utilized NAD^+^/NADH Assay Kit with WST‐8 (Beyotime, #C3601) according to manufacturer's protocols, and the statistical methods of the ratio of NADH/NAD^+^ were provided in manufacture's protocols. The mitochondria isolations and verifications were conducted using Cell Mitochondria Isolation Kit (Beyotime, #C3601) according to manufacturer's guidelines. With respect to the activity assessment of RCC IV we used freshly isolated mitochondria in accordance to a previously reported method (Kirby et al., 2007). For FACs analysis, cells were respectively detected using ROS probe (CM‐H2DCFDA, InvitrogenTM, ThermoFisher) and Mitochondrial membrane potential assay kit with JC‐1 (Betotime, #C2006). And the flowcytometry was carried out using Attune NxT Flow Cytometer (Attune NxT3, nvitrogenTM, ThermoFisher).

### 4.6 Transcription analysis

All RT‐qPCR data in this study used the mRNAs which extracted from freshly isolated mitochondria. mRNAs were extracted using the TRIzol™ (Invitrogen, ThermoFisher). Complementary DNA was synthesized by using the HiScript III RT SuperMix for qPCR (Vazyme). Then quantitative real‐time PCR was performed in triplicate by using AceQ Universal SYBR qPCR Master Mix (Vazyme) for PCR reactions on an iCycler Real‐Time Detection System (BioRad, USA). The relative amount of mRNA was normalized to mitochondrially encoded NADH dehydrogenase 2(MT‐ND2). Primers used for RT‐qPCR are listed in Extended Data Table 2. The semi‐quantitative agarose gel electrophoresis was conducted using 2% agarose gel (TsingKe, #9012‐36‐6, China).

### 4.7 Immunofluorescence imsenescence

For golden‐standard anti‐DNA G4 antibody (Merck Millipore, #MABE1126, clone 1H6) detection, cells were fixed in 4% formaldehyde/PBS for 10 min and then were treated with 0.1% Triton X‐100/PBS for 15 min. After blocking w/ 5%BSA in PBST for 20 min, then cells were incubated with corresponding first antibodies overnight. At the second day cells were washed in fresh PBS three times and then were incubated using specific second antibody (Cy3‐conjuncted goat anti‐mouse second antibody, Boster, Cat#BA1031) and DAPI for 2 h at room temperature. Finally, cells were washed using PBS for three times at 10 min per time, mounted using anti‐fluorescence fate mount medium, and detected.

### 4.8 Statistics and image processing

For comparisons of multiple groups One‐way ANOVA with post‐hoc Bonferroni correction was carried out, and Student's t test was performed to determine the statistical significance between two groups. Histograms were showed as mean ± SD (standard deviations). For statistical significance in histograms, labels were defined as follows, * is *p* < 0.05; ** is *p* < 0.01; and *** is *p* < 0.001. All results in this study were presented in the presence of at least three independent biological experiments. All fluorescent images were processed with Image Pro Plus 6.0 (Media Cybernetics, Rockville, MD, USA) or ImageJ (ImageJ software v1.51w). FACS images were processed via FlowJo 10 (FlowJo LLC, USA).

## 5 RESOURCES AVAILABILITY

### 5.1 Materials Availability

All unique/stable reagents generated in this study are available from Prof. Fanyuan Yu (fanyuan_yu@outlook.com) with a completed Materials Transfer Agreement.

## AUTHOR CONTRIBUTIONS

F.Y., and K.Y. conceived the study; F.Y., K.Y., and F.L. performed research; F.Y., K.Y. and F.L. analyzed data; F.Y. and K.Y. wrote the manuscript; and F.Y. and L.Y. reviewed and edited the manuscript.

## CONFLICT OF INTEREST STATEMENT

The authors declare no conflict of interest.

## Supporting information


Data S1.

## Data Availability

This paper does not report any original code. Any additional information required to reanalyze the data reported in this paper is available from the corresponding authors upon request.
