# Supplementary material for: Accumulation of DNA G‐quadruplex in mitochondrial genome hallmarks mesenchymal senescence
Source: Aging Cell. 2024 Jul 2;23(10):e14265. doi: 10.1111/acel.14265 (PMC11464107; doi:10.1111/acel.14265)
Supplement: Supplementary file 1 — Data S1. [file ACEL-23-e14265-s001.pdf]

Supplemental materials

Accumulation of DNA G-quadruplex in Mitochondrial Genome Hallmarks Mesenchymal Senescence

Kangkang Yu[1, 2, 3, *], Feifei Li[1, *], Ling Ye[1, 4, #], Fanyuan Yu[1, 4, #, *]

1 State Key Laboratory of Oral Diseases & National Clinical Research Center for Oral Diseases, West China Hospital of Stomatology, Sichuan University, China
2 Key Laboratory of Green Chemistry and Technology (Ministry of Education), College of Chemistry, Sichuan University, China
3 Key Laboratory of Bio-resources and Eco-environment (Ministry of Education), College of Life Sciences, Sichuan University, China.
4 Department of Endodontics, West China Hospital of Stomatology, Sichuan University
* These authors contribute equally to this work.
# To whom the correspondence should be addressed: Prof. Ling Ye (yeling@scu.edu.cn) and Prof. Fanyuan Yu (fanyuan_yu@outlook.com).

Extended Data

Accumulation of DNA G-quadruplex in Mitochondrial Genome Hallmarks Mesenchymal Senescence

Kangkang Yu[1, 2, 3, *], Feifei Li[1, *], Ling Ye[1, 4, #], Fanyuan Yu[1, 4, #, *]

1 State Key Laboratory of Oral Diseases & National Clinical Research Center for Oral Diseases, West China Hospital of Stomatology, Sichuan University, China
2 Key Laboratory of Green Chemistry and Technology (Ministry of Education), College of Chemistry, Sichuan University, China
3 Key Laboratory of Bio-resources and Eco-environment (Ministry of Education), College of Life Sciences, Sichuan University, China.
4 Department of Endodontics, West China Hospital of Stomatology, Sichuan University
* These authors contribute equally to this work.
# To whom the correspondence should be addressed: Prof. Ling Ye (yeling@scu.edu.cn) and Prof. Fanyuan Yu (fanyuan_yu@outlook.com).

Extended Data Scheme1 The synthetic routes for TPA-mTO, TPA-QL and TPA-BTA.

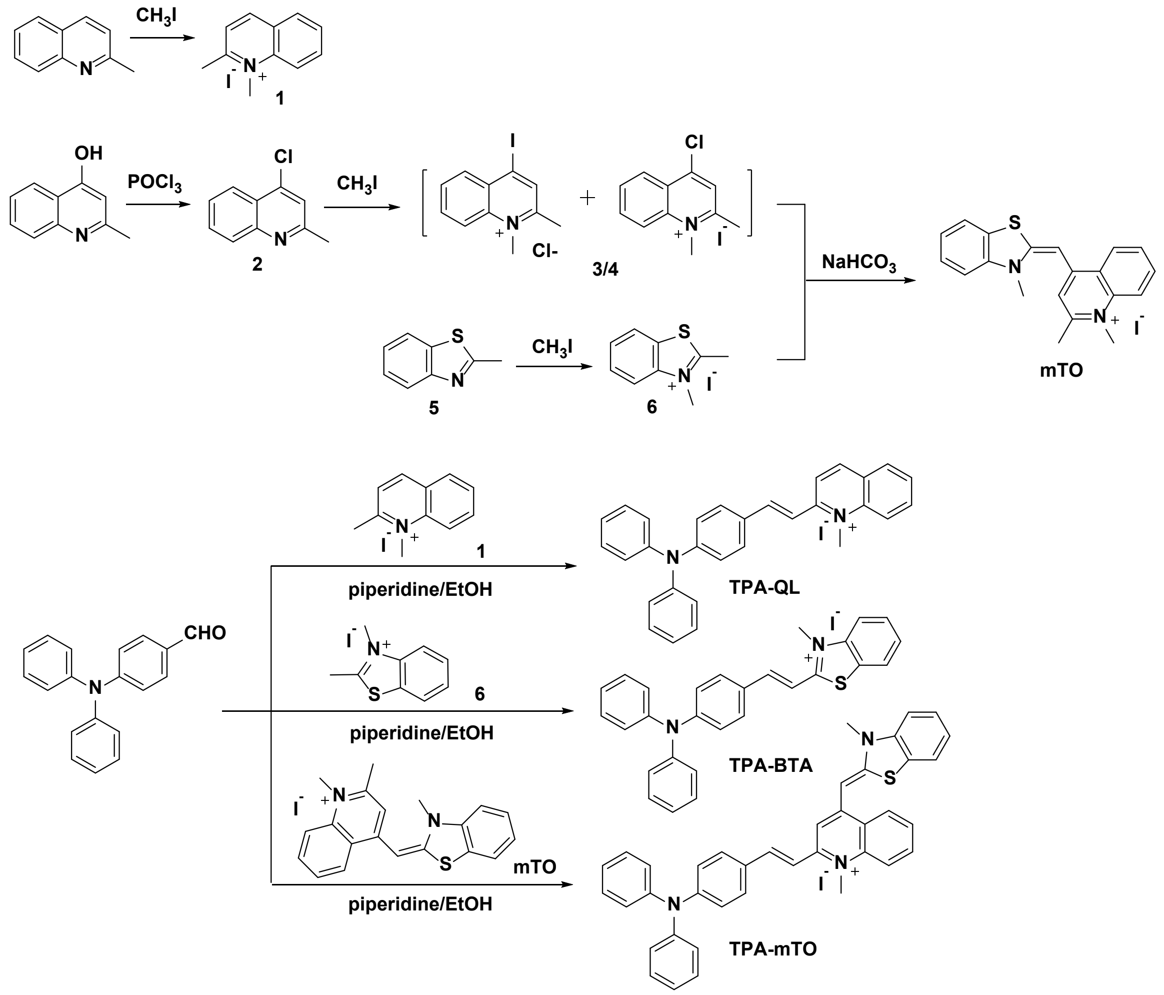

Extended Data Fig.1 Fluorescent properties of the newly developed probes.

a. Molecular orbital amplitude plots of HOMO and LUMO energy levels of TPE-mTO, TPA-mTO, TPA-BTA and TPA-QL calculated through B3LYP/6-31G (d, p) basis set. b and c. The fluorescent intensity of 1 μM probe at 650 nm against the ratio of [nucleic acid sample & probe]/[probe], the testing buffer is 10 mM Tris-HCl (pH 7.4, containing 50 mM KCl), λex = 488 nm. d. The cytotoxicity of TPA-mTO. e. The distributions of TPA-QL and TPA-TBA in A549 cells. The cells respectively stained with 1 μM TPA-QL and TPA-TBA for 15 min and wish with PBS, then incubated the cells with 1 μM MitoTracker Green (MTG) for 20 min. Ex@488 nm for the MTG channel (500-530 nm), Ex@543 nm for TPA-mTO channel (600-700 nm). Bars: 5 μm. f. The distributions of TPA-mTO in A549 cells. The cells were stained with 1 μM TPA-mTO for 15 min and wish with PBS, then incubated the cells with 1 μM LysoTracker Green (LTG) for 20 min. Ex@488 nm for the MTG and LTG channel (500-530 nm), Ex@543 nm for TPA-mTO channel (600-700 nm). Bars: 10 μm

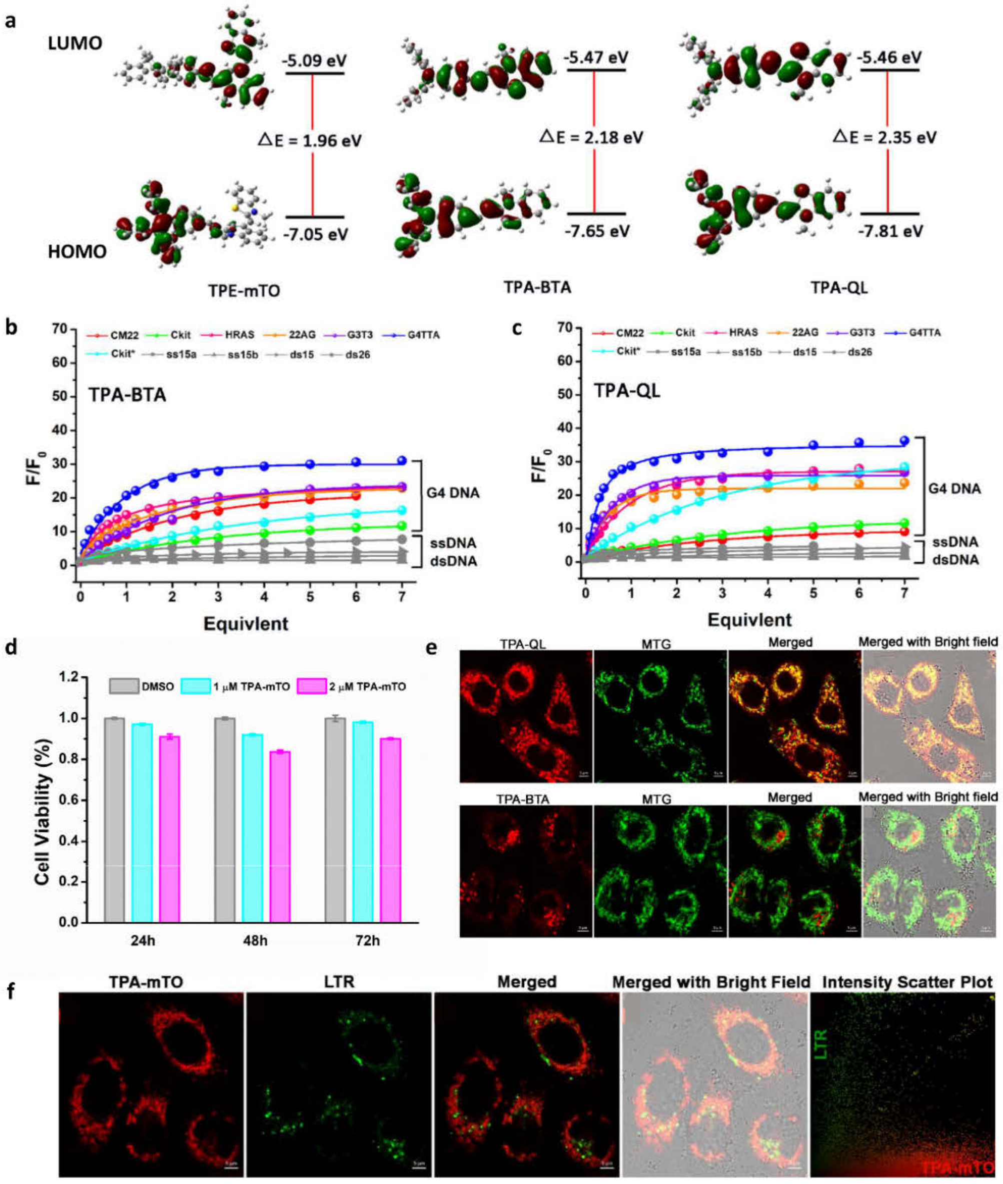

Extended Data Table 1 Sequence of oligonucleotides used in the present study.

Name	Sequence (from 5' to 3')	Structure in 10 mM Tris-HCl Buffer (contain 50 mM K_+)
CM22	TGAGGGTGGGTAGGGTGGGTAA	Parallel G-quadruplex
Ckit	AGGGAGGGCGCTGGGAGGAGGG	Parallel G-quadruplex
HRAS	TCGGGTTGCGGGCGCAGGGCACGGGCG	Antiparallel G-quadruplex
22AG	AGGGTTAGGGTTAGGGTTAGGG	Hybrid-Type G-quadruplex
G3T3	GGGTTTGGGTTTGGGTTTGGG	Hybrid-Type G-quadruplex
G4TTA	TTAGGGTTAGGGTTAGGGTTAGGG	Hybrid-Type G-quadruplex
Ckit*	GGCGAGGAGGGGCGTGGCCGGC	Hybrid-Type G-quadruplex
ss15a	CGC GCG TTT CGC GCG	Single-Strand DNA
ss15b	CGC GCG AAA CGC GCG	Single-Strand DNA
ds15	ss15a/ss15b	Double-Strand DNA
ds26	CAATCGGATCGAATTCGATCCGATTG	Double-Strand DNA

Extended Data Fig. 2 Imaging of TPA-mTO in live cells.

a. Representative confocal fluorescent images of different cells stained with 1 μM TPA-mTO for 15 min. b. Confocal fluorescent images of different fixed cells stained with 1 μM TPA-mTO and TPE-mTO for 15 min, respectively. c. Fluorescent intensity change of A549 cells separately stained with 1 μM TPA-mTO and 1 μM TPE-mTO with the number of scans of laser irradiation (100% laser power). d. Representative c onfocal fluorescent images of A549 cells after staining for 30 min with 1 μM TPA-mTO and incubating for another 6h, 24h, 48h, and 72h hours in full medium. e. Quantification of the fluorescence enhancement caused by the binding between TPA-mTO and mtG4 in d. f. Representative confocal microscopy of different cells that respectively stained with 1 μM TPA-mTO for 30min with and without PDS. Ex@543 nm for TPA-mTO channel (600-700 nm). Ex@488 nm for the green channel of TPE-mTO (Em@500-600 nm).

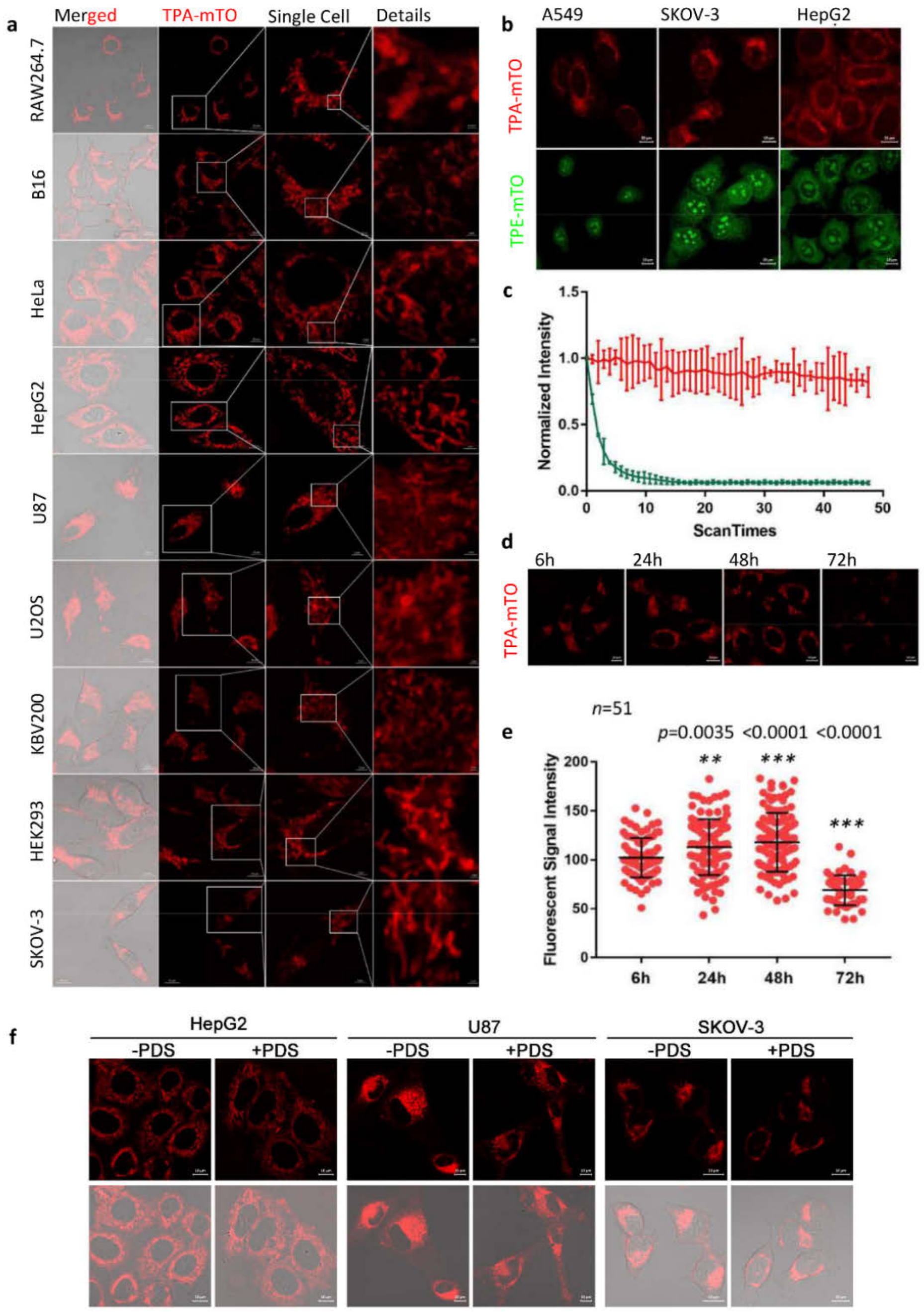

Extended Data Fig. 3 Evidences of MSCs senescence. a. Sa-β-gal staining of young and senile MSCs and according quantification data. b. EdU incorporation labelling and its statistic summary.

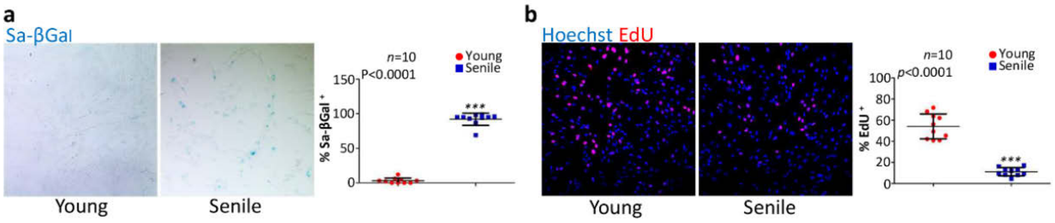

Extended Data Table 2 Sequence of mtDNA that might form G4 structure.

Name	Sequence
MT-ND6-1	GGTTTAGTAATGGGGTTTGTGG
MT-ND6-2	GGTTTAGTATTGATTGTTAGCGGTGTGGTCGGG
MT-ND6-3	TTTTGGGGGAGGTTATATGGGTTT
MT-ND6-4	GGGGGAATGATGGTTGTCTTTGG
MT-ND6-5	GGCATGGGGGTCAGGGGTTGAGG
MT-ND6-6	GATGGAGGTAGGATTGGTGC
MT-ND6-7	ATGATGGGGTGGTGGTTGTGG
MT-ND6-8	GGGGTCAGGGTTGATTCGGGAGGATCCTATTGGTGCGGG
MT-ND6-9	GGTGTATATATTGTAATTGAGATTGCTCGGGGGAATAGG
MT-RNR1-1	GGACCTGGCGGTGCTTCATATCCCTCTAGAGG
MT-RNR1-2	GGTCAAGGTGTAGCCCATGAGGTGGCAAGAAATGG
MT-RNR1-3	GGAGACAAGTCGTAACATGGTAAGTGTACTGGAAAGTGCACTTG
MT-RNR2-1	GGGAAGATTTATAGGTAGAGGCGACAAACCTACCGAGCCTGG
MT-RNR2-2	GGCATGCTCATAAGGAAAGGTTAAAAAAAGTAAAAGGAACTCGG
MT-RNR2-3	GGTTGGGGCGACCTCGG
MT-RNR2-4	GGATCAGGACATCCCGATGGTGCAGCCGCTATTAAAGG
MT-CO1-1	GGAACACTATACCTATTATTCGGCGCATGAGCTGGAGTCCTAGG
MT-CO1-2	GGAGGCTTTGGCAACTGACTAGTTCCCCTAATAATCGG
MT-CO1-3	GGAGGCCGGAGCAGGAACAGG
MT-CO1-4	GGAAAAAAAGAACCATTTGGATACATAGGTATGG
MT-CO1-5	GGATTCATCTTTCTTTTCACCGTAGGTGGCCTGACTGG
MT-CO3-1	GGAGGGCACTGGCCCCCAACAGG
MT-CO3-2	GGCATCTACGGCTCAACATTTTTTGTAGCCACAGGCTTCCACGG
MT-TY	GGCTGAGTGAAGCATTGGACTGTAAATCTAAAGACAGGGGTTAGG
MT-TQ	GGGGTGTGATAGGTGGCACGGAGAATTTTGGATTCTCAGGGATGG
MT-TL2	GGATAACAGCTATCCATTGGTCTTAGGCCCCAAAAATTTTGG
MT-TS1	GGAGGCCATGGGGTTGG

Supplementary Information

Accumulation of DNA G-quadruplex in Mitochondrial Genome Hallmarks Mesenchymal Senescence

Kangkang Yu[1, 2, 3, *], Feifei Li[1, *], Ling Ye[1, 4, #], Fanyuan Yu[1, 4, #, *]

1 State Key Laboratory of Oral Diseases & National Clinical Research Center for Oral Diseases, West China Hospital of Stomatology, Sichuan University, China
2 Key Laboratory of Green Chemistry and Technology (Ministry of Education), College of Chemistry, Sichuan University, China
3 Key Laboratory of Bio-resources and Eco-environment (Ministry of Education), College of Life Sciences, Sichuan University, China.
4 Department of Endodontics, West China Hospital of Stomatology, Sichuan University
* These authors contribute equally to this work.
# To whom the correspondence should be addressed: Prof. Ling Ye (yeling@scu.edu.cn) and Prof. Fanyuan Yu (fanyuan_yu@outlook.com).

1. Synthesis and Characterization

1.1 Materials and instruments

^{1}H NMR and ^{13}C NMR spectra were measured on a Bruker AM400 NMR spectrometer. Proton chemical shifts of the NMR spectra are given in ppm relative to an internal reference, TMS (1H, 0.00 ppm). ESI-MS and HRMS spectral data were recorded on a Finnigan LCQDECA and a Bruker Daltonics Bio TOF mass spectrometer, respectively. Fluorescence emission spectra were tested using a F-7000 Fluorescence Spectrophotometer (HITACHI) at 298 K. The imaging experiments of living cells were performed on a ZEISS LSM 780 confocal laser scanning microscope (CLSM). Circular dichroism studies were carried out on a Chirascan^{TM}-plus ACD Circular Dichroism Spectrometer. The gel electrophoresis results were photographed by C600 bioanalytical imaging system (Azure Biosystems).

All the commercially available chemicals that used for synthesis were analytical grade and used without further purification, unless otherwise noted. All the solvents were either HPLC or spectroscopic grade in the optical spectroscopic studies. The commercial dyes that used for labeling intracellular lysosomes and mitochondrion were purchased from Invitrogen, including Lysotracker™ green DND-26 (LTG) and MitoTracker™ Green FM (MTG),. All the oligonucleotides were bought from Sangon Biotechnology Co., Ltd. (Shanghai, China) and the sequences were listed in Table S1. DNase was purchased from Sigma-Aldrich (D4527).

All the oligonucleotides were dissolved in, their concentrations were tested from the absorbance at 260 nm based on the relevant respective molar extinction coefficients, respectively. The G-quadruplex formation of each oligonucleotides were determined by circular dichroism (CD) measurements, the stock solutions of each G-quadruplex (100 μM) were dissolved in 10 mM Tris-HCl buffer (containing 50 mM KCl, pH 7.4) and stored at 4 °C.

1.2 The preparation of TPA-mTO, TPA-QL and TPA-BTA

Compounds **1**, **2**, **3**, **4**, **5**, and **6** (Extended Data Scheme1)were prepared according to our previous work.[1]

Synthesis and Characterization of TPA-QL

4-(diphenylamino) benzaldehyde (136.0 mg, 0.25 mmol) and **1** (142.5 mg, 0.5 mmol) were dissolved in 20 mL dry ethanol, then added 5 drops of piperidine to the mixture. The mixture was refluxed under nitrogen overnight. After cooling down to room temperature, the solvent was evaporated under reduced pressure. The crude product was purified by silica gel column eluting with DCM/ methanol (20:1, v/v) to give 65.6 mg brownish red solid (yield 24.3%). ^{1}H NMR (400 MHz, DMSO-*d6*): δ 8.97 (d, 1H, *J* = 8.0Hz), 8.56-8.50 (dd, 2H, *J* = 8.0Hz), 8.31 (d, 1H, *J* = 6.8Hz), 8.23-8.13 (m, 2H), 7.94-7.85 (m, 3H), 7.73 (d, 1H, *J* = 12.0Hz), 7.42 (t, 4H, *J* = 8.0Hz), 7.23-7.17 (m, 6H), 6.96 (s, 3H, *J* = 8.0Hz), 4.51 (s, 3H) ppm; HRMS: (ESI) m/z calcd for **TPA-QL** $[M - I]^+$: 413.2012, found: 413.2011.

Synthesis and Characterization of TPA-BTA

4-(diphenylamino)benzaldehyde (136.0 mg, 0.5 mmol) and **6** (145.0 mg, 0.5 mmol) were dissolved in 20 mL dry ethanol, then added 5 drops of piperidine to the mixture.

The mixture was refluxed under nitrogen overnight. After cooling down to room temperature, the solvent was evaporated under reduced pressure. The crude product was purified by silica gel column eluting with DCM/ methanol (20:1, v/v) to give 55.3 mg drak red solid (yield 20.2%). ^{1}H NMR (400 MHz, DMSO-*d6*): δ 8.34 (d, 1H, *J* = 8.0Hz), 8.16-8.07 (m, 2H), 7.88 (d, 2H, *J* = 8.0Hz), 7.80 (dd, 2H, *J* = 8.0Hz), 7.71 (t, 1H, *J* = 8.0Hz), 7.40 (t, 4H, 8.0Hz), 7.23-7.16 (m, 6H), 6.88 (d, 2H, *J* = 8.0Hz), 4.25 (s, 3H) ppm; HRMS: (ESI) m/z calcd for **TPA-BTA** [M - I]$^{+}$: 419.1576, found: 419.1577.

Synthesis and Characterization of TPA-mTO

4-(diphenylamino)benzaldehyde (68.0 mg, 0.25 mmol) and **m-TO** (111.5 mg, 0.25 mmol) were dissolved in 20 mL dry ethanol, then added 5 drops of piperidine to the mixture. The mixture was refluxed under nitrogen overnight. After cooling down to room temperature, the solvent was evaporated under reduced pressure. The crude product was purified by silica gel column eluting with DCM/ methanol (20:1, v/v) to give 45.6 mg brownish red solid (yield 31.7%). ^{1}H NMR (400 MHz, DMSO-*d*6): δ 8.62 (d, 1H, J = 7.6Hz), 7.92 (d, 1H, J = 8.0Hz), 7.65 (d, 2H, J = 8.4Hz), 7.56 (d, 1H, J = 8.8Hz), 7.49-7.44 (m, 2H), 7.39-7.27 (m, 6H), 7.21 (t, 2H, J = 8.8Hz), 7.13-7.06 (m, 8H), 6.98 (d, 2H, J = 8.4Hz), 6.54 (s,1H), 4.03 (s, 3H), 3.82 (s, 3H) ppm; ^{13}C NMR (100 MHz, DMSO-*d*6): δ 159.2, 152.3, 149.6, 147.5, 146.7, 140.9, 140.8, 139.2, 133.4, 130.4, 130.2, 128.7, 128.4,126.7, 125.5, 124.8, 124.4, 124.1, 123.7, 123.3, 121.4, 119.1, 118.7, 108.0, 87.9, 38.4, 34.0; HRMS: (ESI) m/z calcd for **TPA-mTO** [M - I]$^{+}$: 574.2311, found: 574.2313.

Fig. S1 a. ^{1}H NMR spectrum and b. ^{13}C NMR spectrum of TPA-mTO. ^{1}H NMR spectrum of c. TPA-QL and d. TPA-BTA.

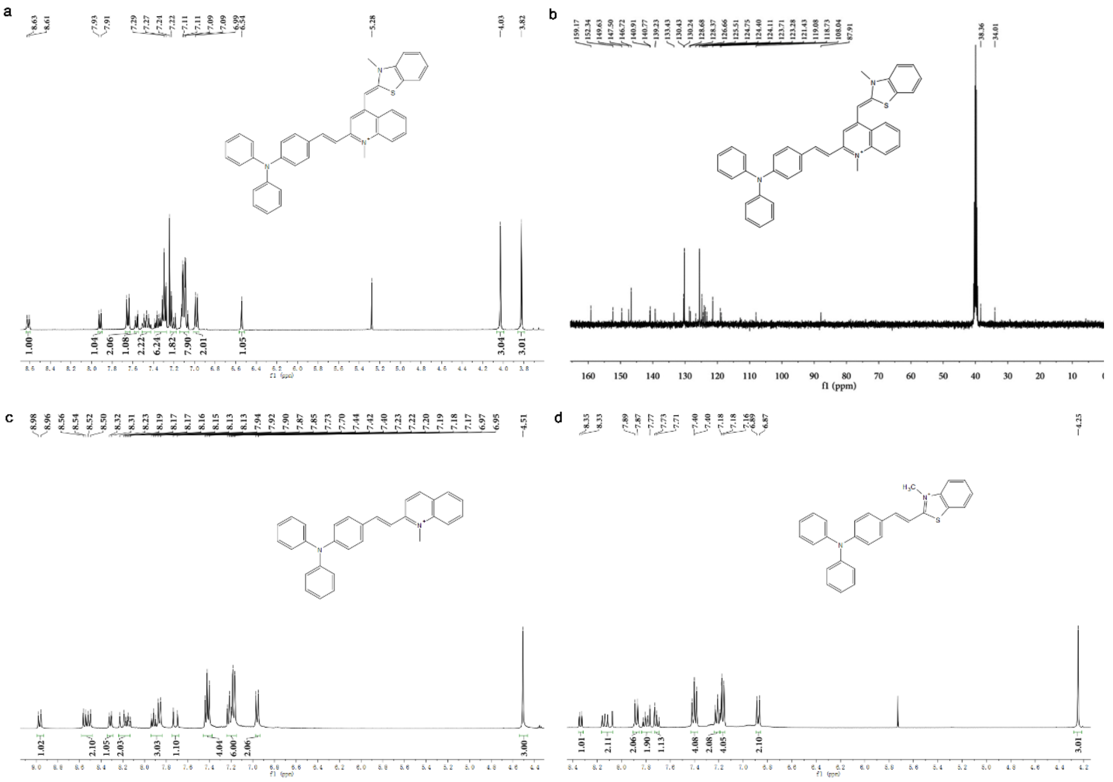

1.3 General methods

Fluorescence measurements

The Fluorescent emission spectra were detected on a HITACHI F-7000 spectrofluorophotometer. 1 mM stock solutions of **TPA-mTO**, **TPA-QL** and **TPA-BTA** were firstly prepared in DMSO. The fluorescence testing buffer was 10 mM Tris-HCl buffer containing 50 mM KCl at pH 7.4. For fluorescent testing, 1 mL **TPA-mTO**, **TPA-QL** or **TPA-BTA** solution (1 μM) was filled in a quartz dish (3.5 mL, optical path length is 1 cm) and the stock solution of different G-quadruplexes was added into the quartz cell using a microsyringe, respectively. The excitation and emission slits of fluorescence spectra were set at 5.0 nm if not specified. After each addition of G-quadruplex, the solution was stirred and equilibrate for at least 5 min.

Calculations of apparent binding constants

The apparent binding constants (K_a) was calculated based on the following equation[2]

$$\frac{F}{F_0} = 1 + \frac{Q-1}{2}\left[A + 1 + x - \sqrt{(A+1+x)^2 - 4x}\right]$$

$$x = \mathrm{n}\frac{C_{G-quadruplexe\ DNA}}{C_{TPA-mTO}}$$

$$\mathrm{A} = \frac{1}{K_a C_{TPA-mTO}}$$

F₀ means fluorescence intensity of **TPA-mTO** only at 650 nm, *F* stands for fluorescence intensity at 650 nm of **TPA-mTO** with different G-quadruplexes, *n* is the putative number of **TPA-mTO** binding to a certain G-quadruplexes. To establish the

fitting curve, Q and A were set up as parameters using the fitting routine in OriginPro 8.5.1 software, whereas n was changed to acquire a better fit.

Calculations of detection limit

The detection limit (LOD) was calculated based on the following equation[3]

$$\mathrm{LOD} = \frac{3\sigma}{\mathrm{k}}$$

σ means the standard deviation of multiple blank measurements, k represents the slope of the calibration curve. (The fluorescence emission of **TPA-mTO** was detected 10 times to obtain the standard deviation of blank measurements.)

Job plots

The stoichiometry between **TPA-mTO** and different G-quadruplexes was assessed by independent experiments using various concentrations of **TPA-mTO** and different G-quadruplexes, while the sum of their concentrations remains the same (1 μM). The fluorescence signal of the mixture of suitable amount of **TPA-mTO** and the correspondent amount of G-quadruplex was measured after 5 min incubation.

The fluorescence intensity of different mixture at the emission wavelength 530 nm were plotted as the function of the input mole fractions of different G-quadruplexes. In the resulting plot, the break point corresponded to the mole fraction of **TPA-mTO** in the complex.

Circular dichroism detection

A quartz dish with a 10 mm path length was used for recording the CD spectra over a wavelength range of 230–350 nm with a 2 nm bandwidth, 0.5 nm step size and time of 100 nm/min. The final CD spectra represented an average of three scans, zero-corrected at 320 nm and normalized (molar ellipticity θ is quoted in 10^5 deg cm2 $dmol^{-1}$). The buffer baseline (10 mM Tris-HCl buffer containing 50 mM KCl at pH 7.4.) was collected in the same quartz dish and it was subtracted from the CD spectra of each sample. Final analysis of the CD spectra was using OriginPro 8.5.1.

Molecular Docking Study

The molecular docking studies were performed using GOLD (Genetic Optimization of Ligand Docking) 5.0.[4] The X-ray crystal structure (PDB entry 2J6M) of the **CM22** G-quadruplex bound to compound quindoline was used as a reference in the docking studies.[5] The Discovery Studio 3.1 (Accelrys, Inc. USA) software package was used to prepare the structure including adding hydrogen atoms, removing water molecules, and assigning force field (here the CHARMm force field was adopted).

Living cells imaging

Cells were cultured in Dulbecco's modified Eagle medium (DMEM) or Roswell Park Memorial Institute 1640 medium (RPMI 1640), which contained 10% foetal bovine serum (FBS) and 1% antibiotic–antimycotic (penicillin−streptomycin, 10000 U/mL) at 37 °C in a 5% CO2/95% air incubator. For fluorescence imaging, cells (4×10^3 per well)

were loaded onto confocal dishes and incubated for 24 h. Before starting the staining experiments, the cells were washed once with PBS (10 mM, pH 7.40).
Group 1: Cells were incubated with 2 μM **TPA-mTO** for 15 min at 37 °C, respectively. Then, the cells did not need to wash and were ready for imaging;
Group 2: Cells were incubated with 2 μM **TPA-QL** for 15 min at 37 °C. Then, the cells did not need to wash and were ready for imaging;
Group 3: Cells were incubated with 2 μM **TPA-BTA** for 15 min at 37 °C. Then, the cells did not need to wash and were ready for imaging;

Co-localization imaging

A549 and HL-7702 cells were cultured in Roswell Park Memorial Institute 1640 medium (RPMI1640) or Dulbecco's modified Eagle medium (DMEM), which contained 10% foetal bovine serum (FBS) and 1% antibiotic–antimycotic (penicillin−streptomycin, 10000 U/mL) at 37 °C in a 5% CO2/95% air incubator. For fluorescence imaging, cells (4×10^3 per well) were loaded onto confocal dishes and incubated for 24 h. Before starting the staining experiments, the cells were washed once with PBS (10 mM, pH 7.40).
Group 1: A549 and HL-7702 cells were incubated with 2 μM **TPA-mTO** for 15 min at 37 °C, respectively. Then, Lyso Tracker Green (LTG, 1 μM) was added, and the cells were incubated for another 20 min. The confocal fluorescence images were captured after washing the cells twice with PBS.
Group 2: A549 and HL-7702 cells were incubated with 2 μM **TPA-mTO** for 15 min at 37 °C, respectively. Then, Mito Tracker Green (MTG, 1 μM) was added, and the cells were incubated for another 20 min. The confocal fluorescence images were captured after washing the cells twice with PBS.
Group 3: A549 cells were incubated with 2 μM **TPA-QL** for 15 min at 37 °C, respectively. Then, Lyso Tracker Green (LTG 1 μM) was added, and the cells were incubated for another 20 min. The confocal fluorescence images were captured after washing the cells twice with PBS.
Group 4: A549 cells were incubated with 2 μM **TPA-BTA** for 15 min at 37 °C, respectively. Then, Mito Tracker Green (MTG 1 μM) was added, and the cells were incubated for another 20 min. The confocal fluorescence images were captured after washing the cells twice with PBS.

Cell imaging without and with DNase treatment

A549 cells (4×10^3 per well) were loaded on confocal dishes and incubated for 24 h. Before the staining experiments, A549 cells were fixed by precooled methanol (-4 °C) for 1 min and washed twice with PBS (10 mM). In order for DNase to pass through the cell membrane, the cells were cultured with 1% Triton X-100 (1 mL) for 2 min and washed twice with PBS.
Group 1: The pretreated A549 cells were stained with 1 μM **TPA-mTO** for 15 min at 37 ℃. Then, the dish was treated with only PBS;
Group 2: The pretreated A549 cells were stained with 1 μM **TPA-mTO** for 15 min at 37 ℃. Then, the dish was treated with 70 ug/mL DNase (37 ℃, 2 h).

Equal parameters and exposure time were used for the both groups.

Fig. S2 Fluorescence titration of TPA-mTO with various oligonucleotides (G-quadruplex: CM22, Ckit, HRAS, 22AG, G3T3, G4TTA, and Ckit*; single-stranded DNA: ss15a and ss15b; double-stranded DNA: ds15 and ds26) in 10mM Tris-HCl buffer, 50mM KCl, pH 7.4. λ_{ex} = 550 nm.

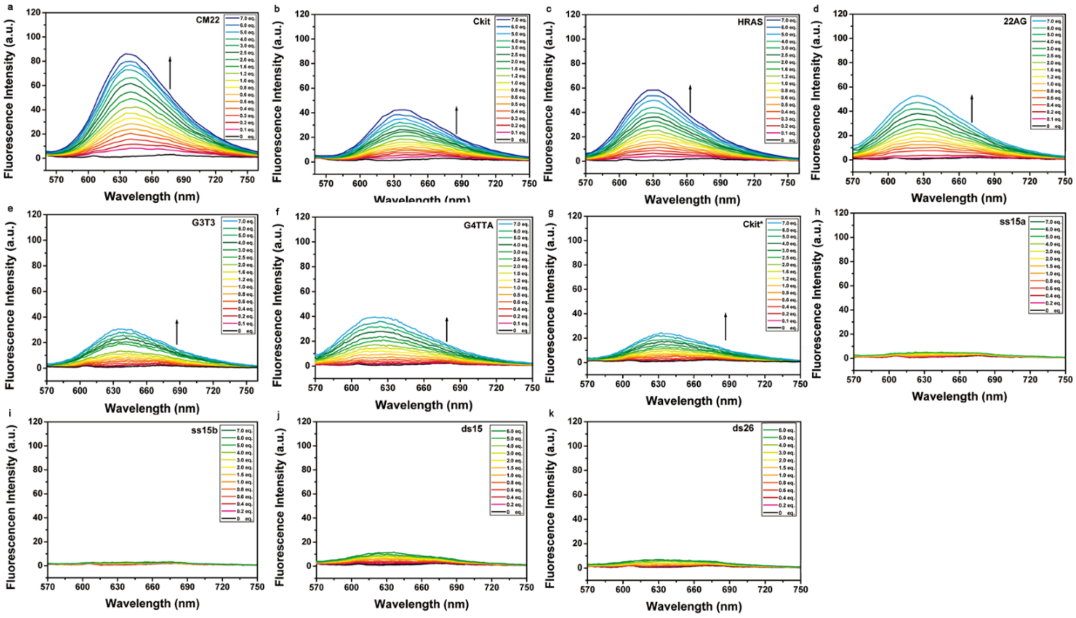

Fig. S3 Fluorescence titration of TPA-QL with various oligonucleotides (G-quadruplex: CM22, Ckit, HRAS, 22AG, G3T3, G4TTA, and Ckit*; single-stranded DNA: ss15a and ss15b; double-stranded DNA: ds15 and ds26) in 10mM Tris-HCl buffer, 50mM KCl, pH 7.4. λ_{ex} = 550 nm.

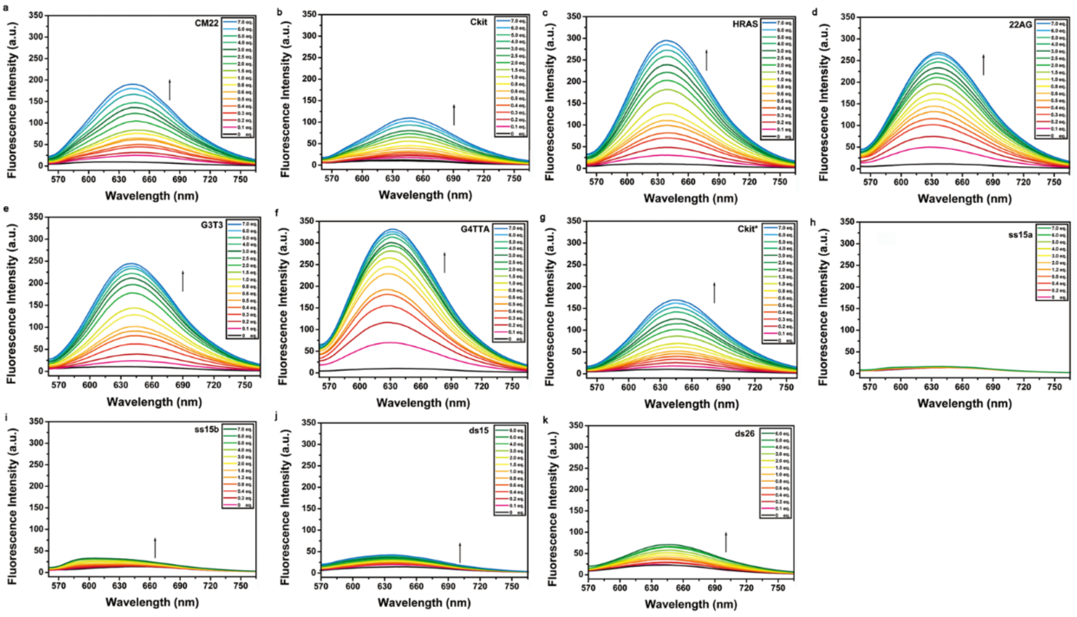

Fig. S4 Fluorescence titration of TPA-BTA with various oligonucleotides (G-quadruplex: CM22, Ckit, HRAS, 22AG, G3T3, G4TTA, and Ckit*; single-stranded DNA: ss15a and ss15b; double-stranded DNA: ds15 and ds26) in 10mM Tris-HCl buffer, 50mM KCl, pH 7.4. λ_{ex} = 550 nm.

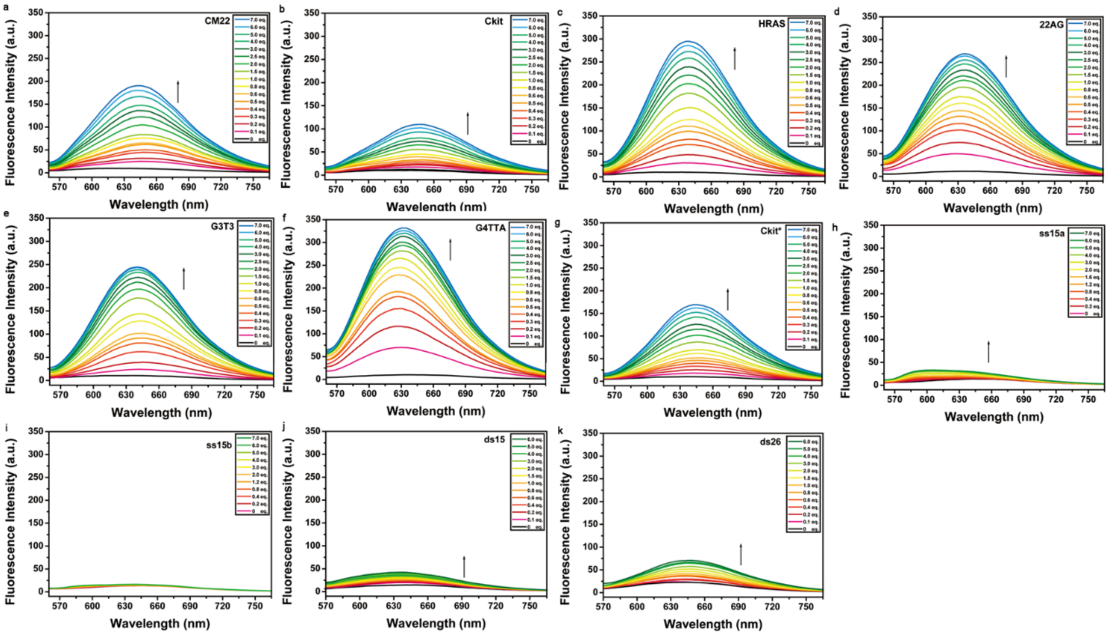

Fig. S5 CD spectra of TPA-mTO with different G-quadreplex-forming oligonucleotides (CM22, Ckit, HRAS, 22AG, G3T3, G4TTA, and Ckit*) in 10 mM Tris-HCl buffer (pH 7.4, 50 mM KCl).

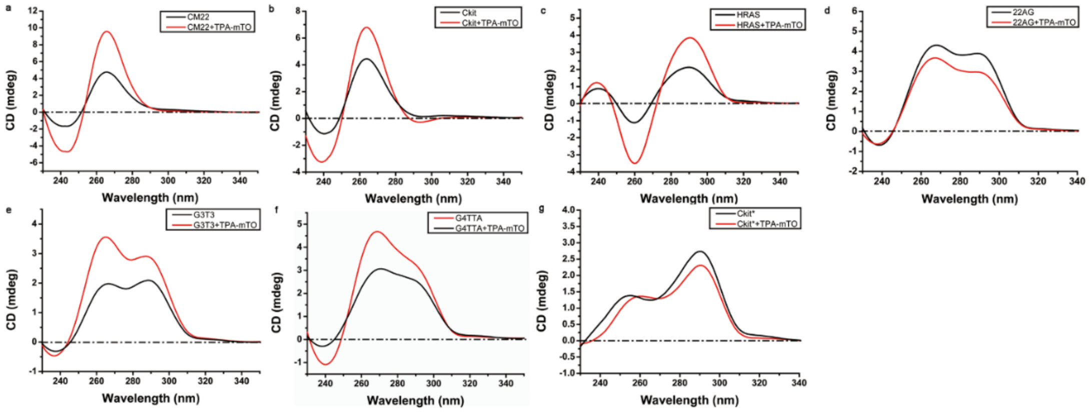

Fig. S6. Calculations of the apparent binding constants of TPA-mTO binging to different G-quadruplexs. 1 μM TPA-mTO in 10 mM Tris-HCl buffer (pH 7.4, 50 mM KCl). the lines represent fitting to the independent-site model.

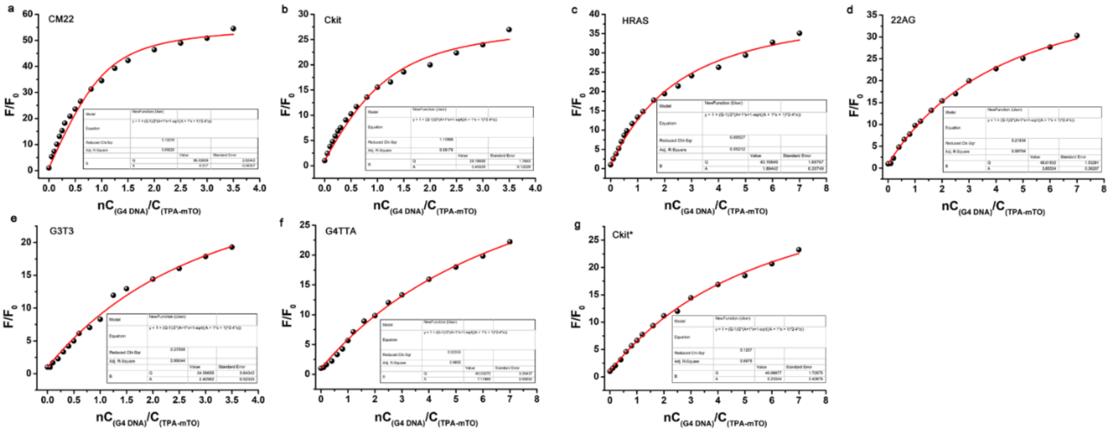

Fig. S7 The linear detection range of TPA-mTO (1 μM) towards different G-quadruplexs.

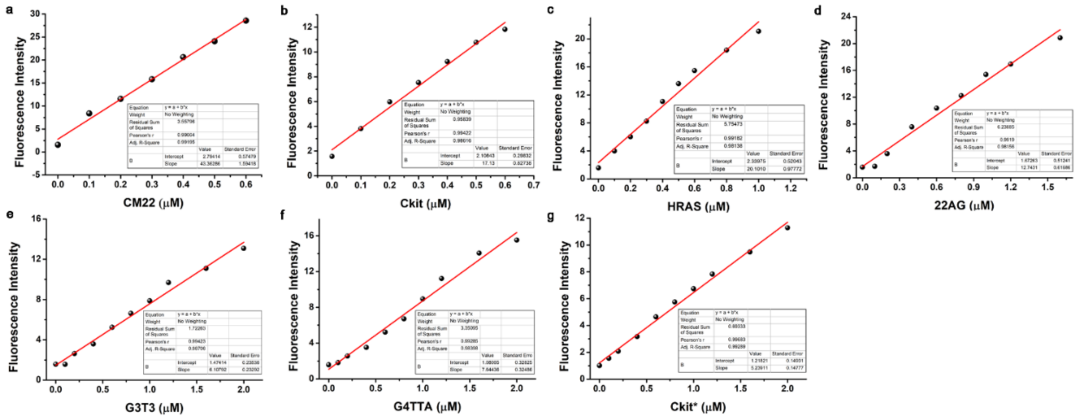

Fig. S8 Job's plot of TPA-mTO and various G-quadreplexes. The total concentration of TPA-mTO and G-quadreplex were kept at 1 μM in 10 mM Tris-HCl buffer (pH 7.4, 50 mM KCl).

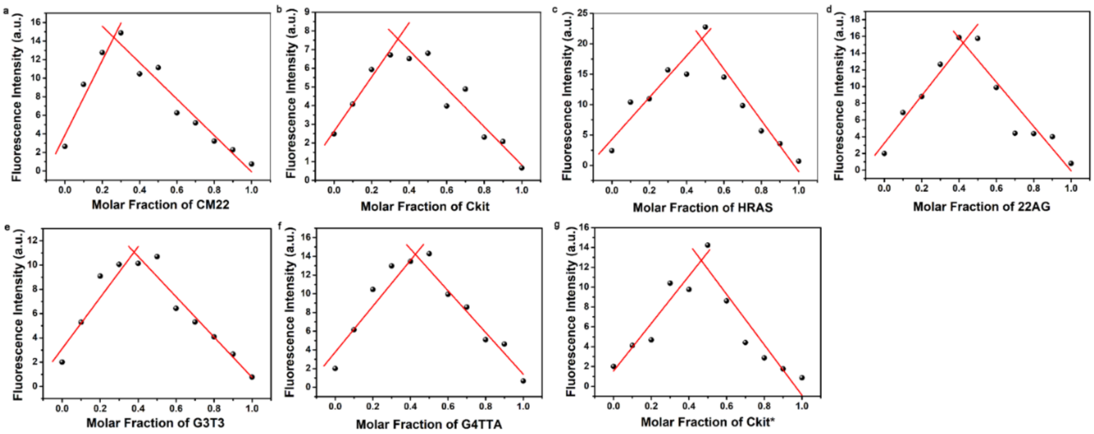

Fig. S9 CD spectra of the selected oligonucleotides of mtDNA in 10 mM Tris-HCl buffer containing 50 mM KCl (black line) and in water (red line). The concentration of oligonucleotides was 3 μM.

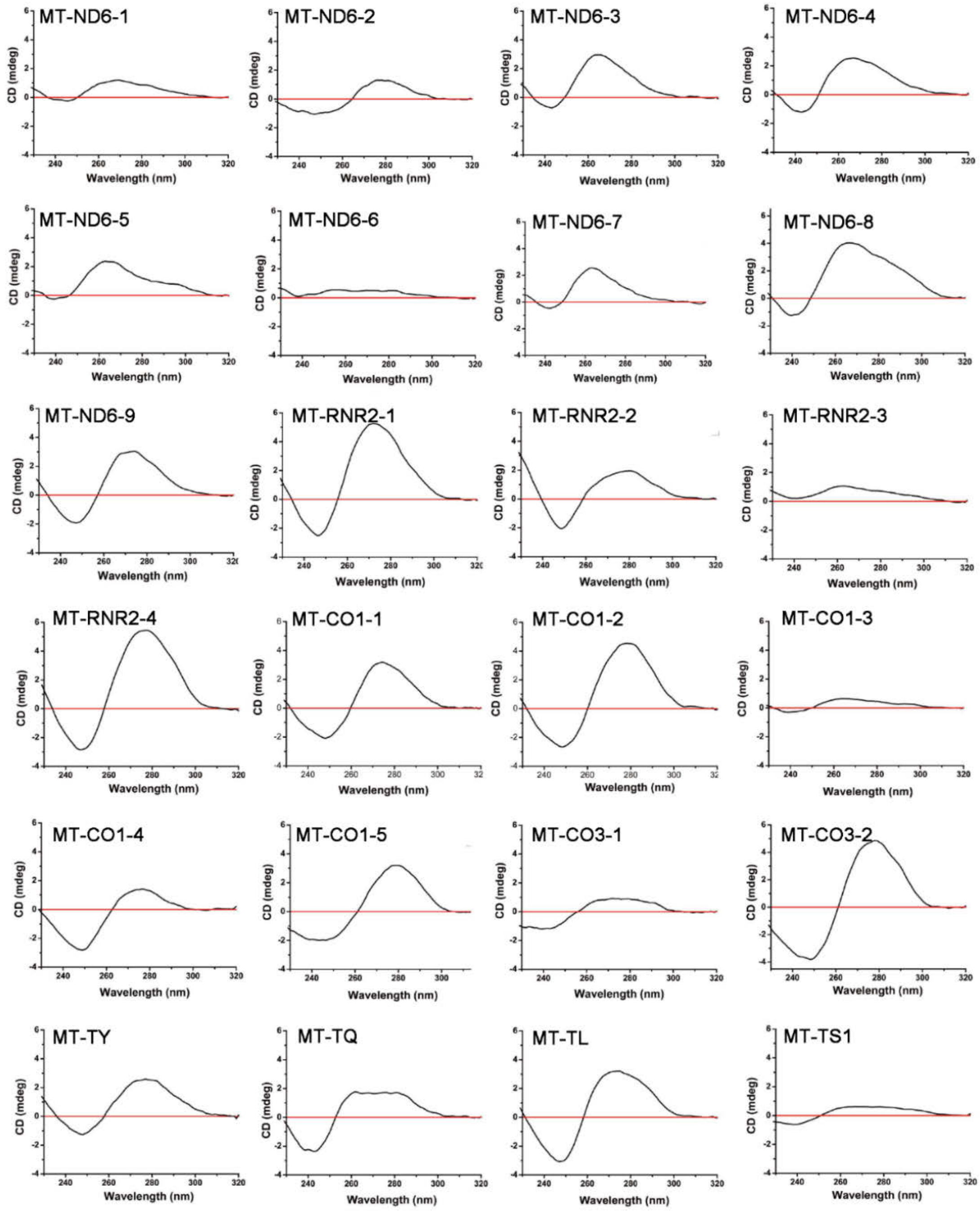

Fig. S10 (a) quantification data of Fig 2j; (b) quantification data of Fig 2k; (c) quantification data of Fig 4a, relating to FM group; (d) quantification data of Fig 5, relating to Young FM group; (e) quantification data of Fig 6f, relating to Young NC group; (f) quantification data of Fig 6g, relating to Senile NC group; ns, no significance; ***, *p*<0.001.

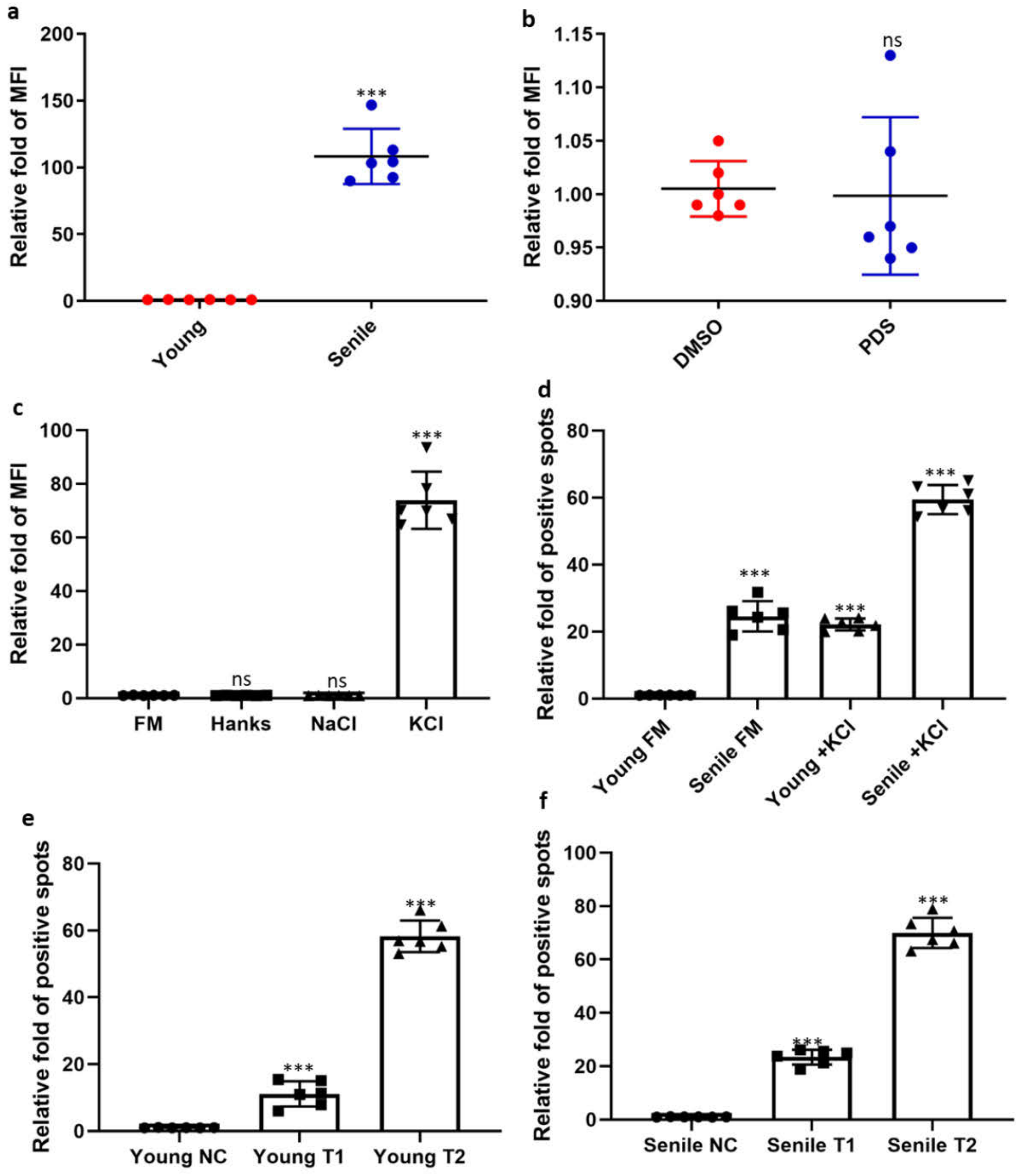

References

1. Li, L., Xu, H., Li, K., Yang, Q., Pan, S., and Yu, X.(2019) Mitochondrial G-quadruplex targeting probe with near-infrared fluorescence emission. Sensor. Actuat. B Chem. 286, 575-582.
2. Wang, M., Ren, G., Zhao, S., Lian, G., Chen, T., Ci, Y., and Li, H. (2018) Development of a carbazole-based fluorescence probe for G-quadruplex DNA: the importance of side-group effect on binding specificity. Spectrochim. Acta. A 199, 441-447.
3. Datta, B.K., Mukherjee, S., Kar, C., Ramesh, A. and Das, G. (2013) Zn^{2+} and pyrophosphate sensing: selective detection in physiological conditions and application in DNA-based estimation of bacterial cell numbers. Anal. Chem. 85, 8369-8375.
4. Jones, G., Willett, P., Glen, R.C., Leach, A.R., and Taylor, R. (1997) Development

and validation of a genetic algorithm for flexible docking. J. Mol. Biol. 267, 727-748.

5. Dai, J., Carver, M. Hurley, L., and Yang, D. (2011) Solution structure of a 2: 1 quindoline-c-MYC G-quadruplex: insights into G-quadruplex-interactive small molecule drug design. J. Am. Chem. Soc. 133, 17673-17680.
